# Pulse Pressure Impairs Cognition via White Matter Disruption

**DOI:** 10.1161/HYPERTENSIONAHA.124.24543

**Published:** 2025-07-10

**Authors:** Deborah L.O. King, Richard N. Henson, Marta Correia, James B. Rowe, Cam-CAN Consortium, Kamen A. Tsvetanov

**Affiliations:** 1 Department of Clinical Neurosciences (D.L.O.K., J.B.R., K.A.T.), University of Cambridge, United Kingdom.; 2 Department of Psychology (D.L.O.K., K.A.T.), University of Cambridge, United Kingdom.; 3 Department of Psychiatry, (R.N.H., M.C.), University of Cambridge, United Kingdom.; 4 Medical Research Council Cognition and Brain Sciences Unit, Cambridge, United Kingdom (R.N.H., M.C., J.B.R., Cam-CAN Consortium).; 5 Cambridge Centre for Ageing and Neuroscience (Cam-CAN), University of Cambridge and MRC Cognition and Brain Sciences Unit, Cambridge, United Kingdom (R.N.H., J.B.R., Cam-CAN Consortium).; 6 Behavioural and Clinical Neuroscience Institute, Cambridge, United Kingdom (J.B.R.).

**Keywords:** aged, blood pressure, cognition, heart rate, risk factors

## Abstract

**BACKGROUND:**

In older adults, elevated pulse pressure predicts cognitive decline, independent of overall blood pressure. It is proposed to compromise cerebrovascular integrity, potentially leading to brain damage, though the underlying mechanisms remain unclear. We hypothesized that pulse pressure affects cognition by disrupting white matter microstructure, and that it does so independently of other cardiovascular risk factors.

**METHODS:**

Latent indices of pulse pressure, overall blood pressure, and heart rate variability were estimated in a cross-sectional, population-based cohort (n=708, aged 18–88 years). An indicator of white matter microstructure was derived from diffusion-weighted imaging, termed the peak width of skeletonized mean diffusivity (PSMD). Cognitive function was assessed using measures of processing speed.

**RESULTS:**

In robust regression, pulse pressure was significantly associated with PSMD, with PSMD also being associated with processing speed. Thus, higher pulse pressure was associated with greater white matter disruption, which in turn was associated with slower processing. This motivated testing whether PSMD mediates the effects of pulse pressure on processing speed using structural equation models. PSMD mediated this effect, accounting for 72% of the effect after adjusting for age, and remained significant after adjusting for other cardiovascular factors. We then expanded the model to show that vascular-related changes in processing speed also drive changes in higher cognitive functions.

**CONCLUSIONS:**

High pulse pressure disrupts the microstructural integrity of white matter in the brain, leading to slower processing speed. We propose that better management of pulse pressure could help to preserve white matter integrity and reduce cognitive decline in later life.

**NOVELTY AND RELEVANCE**

**What Is New?**

High pulse pressure is a commonly overlooked indicator of cardiovascular health. It disrupts the microstructural integrity of white matter in the brain, leading to slower processing speed, in otherwise healthy adults. The effects of pulse pressure were independent of other vascular factors and were consistent across different age groups.

**What Is Relevant?**

The findings highlight how pulse pressure impacts cerebrovascular and cognitive function, providing insights into age-related cognitive decline and promoting healthy cognition throughout the lifespan.

**Clinical/Pathophysiological implications?**

We propose that managing pulse pressure will help preserve white matter integrity and reduce cognitive decline in later life.

With increasing age, arteries lose elasticity and stiffen, leading to increased pulse pressure, the difference between systolic and diastolic blood pressure. The resulting elevated pulse wave velocity and augmented wave reflection allow pulsatile energy to penetrate the cerebral microcirculation.^1^ This pulsatile stress is hypothesized to damage small cerebral vessels, triggering a cascade of events,^2^ which leads to impaired cerebral blood flow and hypoxia. White matter is particularly vulnerable to hypoxia,^3^ due to its narrow, widely spaced arterioles, which are less efficient at maintaining perfusion than those in gray matter.^4–7^ Hypoxia also impairs astrocytes and oligodendrocytes, hindering white matter myelin. White matter aging is associated with demyelination, cell death, and macrostructural changes detectable as lesions and hyperintensities on magnetic resonance imaging, which are linked to cognitive impairment, including vascular dementia. Studies have associated higher pulse pressure with early white matter microstructure changes in middle and old age,^8–11^ suggesting that white matter microstructure mediates the relationship between pulse pressure and cognition, independently of other cardiovascular factors.^12^

Diffusion-weighted imaging provides measures of white matter microstructure, such as mean diffusivity and fractional anisotropy, which reflect water movement. Healthy tissues restrict water flow more than aging tissues, where axonal integrity degrades and perivascular spaces widen. However, averaging diffusion-weighted imaging values across white matter voxels is a common approach that sacrifices sensitivity to tissue heterogeneity. Individuals with a wide range of diffusion values may have focal damage, for example, hyperintensities, even if their mean mean diffusivity/fractional anisotropy values are similar to others.

An alternative approach is to quantify the peak width distribution of skeletonized mean diffusivity (PSMD),^13^ representing the spread of values across the 90% of voxels within the 5th and 95th percentiles.^13^ PSMD increases nonmonotonically with age, accelerating after 60 years of age,^14^ suggesting it may be an early marker of individual aging variations.^14,15^ PSMD associates more strongly with markers of ischemic than neurodegenerative processes^13,16^ and is higher in individuals with cerebrovascular disease than healthy controls.^15^ While PSMD has been linked to clinical status in diabetes and hypertension, it has not been related to continuous vascular health measures—such as pulse pressure—in healthy aging.^17,18^ Understanding what drives the increase in PSMD with age, and whether it mediates the link between vascular factors and cognitive aging, is important.^12^

White matter health is strongly linked to cognition,^19^ with PSMD outperforming established markers of white matter injury in its association with cognitive performance.^13,16,20,21^ The cognitive domain that relates to PSMD most consistently is processing speed.^13,20–23^ This may be due to the role of white matter tracts in transmitting information rapidly. Processing speed is one of the first cognitive domains to decline with age and is thought to underlie declines in other cognitive domains.^24–26^ Previous studies showed that individual differences in white matter were associated with differences in processing speed, which in turn linked to variance in fluid intelligence.^27^ Fluid intelligence declines sharply with age and is more strongly associated with elevated pulse pressure than overall blood pressure or heart rate variability.^12^ These findings support the hypothesis that white matter integrity mediates the relationship between pulse pressure and cognition, particularly processing speed and fluid intelligence.

Here, we tested whether pulse pressure affects cognition through PSMD, using data from a large-scale, lifespan population-based cohort of healthy adults. We hypothesized that pulse pressure affects processing speed via PSMD, even after accounting for other key indicators of vascular health such as global blood pressure levels and heart rate variability. Secondary hypotheses included that (1) the mediation effect increases with age and (2) it extends to fluid intelligence. To test these hypotheses, we used 3 latent vascular factors—pulse pressure, steady-state blood pressure and heart rate variability^12^—to better estimate underlying vascular constructs.^28–31^ We tested these hypotheses in a 3-step process: (1) developing linear and mediation models; (2) repeating these models across age subgroups; and (3) expanding the models from processing speed to fluid intelligence.

## Methods

### Data Availability

To minimize the possibility of unintentionally sharing information that can be used to re-identify private information, a subset of the data generated for this study are available at https://github.com/DebsKing/Pulse_pressure_impairs_cognition_via_white_matter_disruption.git and can be accessed at https://camcan-archive.mrc-cbu.cam.ac.u/dataaccess/.

The raw data are available on request from https://camcan-archive.mrc-cbu.cam.ac.uk/dataaccess/. Statistical analyses were performed in R (version 4.0.2) and RStudio.^32^ PSMD was calculated using the release 1.8.2 (https://github.com/miac-research/psmd/releases).^13^ All summary measures and R code for regression models and SEMs are publicly available at: https://github.com/DebsKing/Pulse_pressure_impairs_cognition_via_white_matter_disruption.git.

### Participants

**Figure 1.**
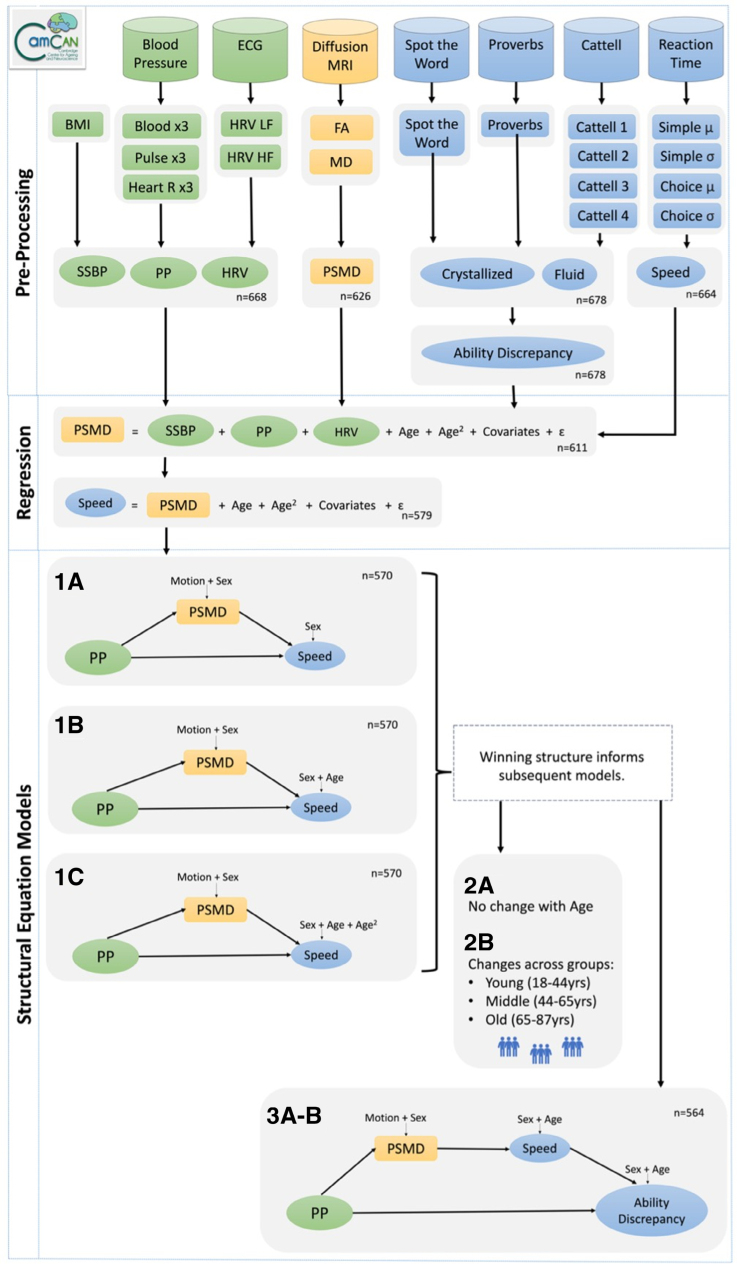
**A schematic representation of the main stages in the data processing and analysis pipeline, to investigate whether peak width of skeletonized mean diffusivity (PSMD), an indicator of white matter disruption, mediated the relationship between pulse pressure (systolic-diastolic) and cognitive decline, in the Cam-CAN data set (n ≤708).** This includes regression models (**middle**) and structural equation models (**bottom**) with simple mediation models (1A–C) informing subsequent models (2A–B and 3A–B).For simplicity, the schematic does not show the quadratic latent vascular factors. Squares represent manifest variables and circles represent latent variables. Symbols: µ, mean; σ, SD. BP indicates blood pressure; BMI, body mass index; FA, fractional anisotropy, Heart R, heart rate; HRV HF, heart rate variability high frequency; HRV LF, heart rate variability low frequency; MD, mean diffusivity; PP, pulse pressure; and SSBP, steady-state blood pressure.

We studied participants in the population-based Cam-CAN (The Cambridge Center for Aging and Neuroscience) cohort, which has deeply phenotyped data on ≈700 adults, aged 18 to 88 years,^33,34^ (see also Supplemental Section A: Methods). Figure 1 illustrates the analytical approach.

### Latent Vascular Factors

The vascular observations were processed following our previous methodology^12^ to construct 3 latent vascular factors: steady-state blood pressure, pulse pressure, and heart rate variability, unless otherwise specified. For more details, see Supplemental Section A: Methods and Figure 1. The pulse pressure factor was the focus of the present theory-driven analysis, but to explore whether pulse pressure acts independently of other vascular signals, the factor scores for all 3 latent vascular factors were extracted and input into the statistical models outlined below. Sensitivity analyses examined whether the results remained consistent when using observed measures of pulse pressure instead of latent vascular factors.

### Cognitive Measures

Assessments of crystallized and fluid intelligence were also outlined previously.^12^ In brief, 6 observed measures were condensed into 2 latent variables, representing crystallized and fluid intelligence (n=678). The difference between these latent cognitive factors was calculated to give the ability discrepancy score.^35^ The ability discrepancy was based on 3 assumptions: (1) fluid and crystallized intelligence measurements are age-invariant; (2) the 2 are highly correlated in youth; and (3) crystallized measures remain stable with age, as previously proposed.^12^

Processing speed was captured through response times in simple and choice tasks in a single latent factor of speed (see Supplemental Section A: Methods).

### Peak Width of Skeletonized Mean Diffusivity

The fractional anisotropy and mean diffusivity maps (see Supplemental Material: Diffusion Tensor Imaging) were used to calculate the global peak width of skeletonized mean diffusivity (PSMD), following previous work^13^ and detailed in Supplemental Section A: Methods.

### Analytical Integration of Vascular, White Matter and Cognitive Measures

Pairwise relationships between vascular, cerebral, and cognitive measures were examined using Pearson’s product-moment correlation coefficients. Since pulse pressure appeared to have a quadratic relationship with PSMD (Figure S6), latent vascular factors were additionally considered in their quadratic forms in the subsequent regression and SEM models. All variables were standardized (mean=0, SD=1) before input into regression and SEM models.

Outliers with undue influence motivated the use of robust linear regression, using the MASS package with default settings.^36,37^ We performed a series of regression models from simple to complex, only retaining variables that benefited model fit. Model fit was investigated using the Akaike Information Criterion, Bayesian Information Criterion, and proportion of variance explained. Results were deemed significant if *P*<0.05. Predictor *P* values were adjusted with Bonferroni corrections for the larger models with over 20 predictors.

Regression models established evidence for relationships between pulse pressure and PSMD, and between PSMD and processing speed (see Supplemental Section A: Linear Regression Models). This motivated formally testing whether the effects of pulse pressure on PSMD drive individual differences in processing speed, that is, whether an association between pulse pressure and processing speed is mediated by PSMD.^38^ This was tested in a series of SEMs using the lavaan package.^39^ Schematic representations of mediation models are shown in Figure 1. The base model directly connects the predictor (green) on the left to the dependent variable (blue) on the right. The diagonal paths are referred to as a and b, which together form the indirect mediating pathway (a×b). The total modeled effect of the predictor on the outcome is denoted as c, while after accounting for the mediating pathway, the remaining variance in the direct path is denoted as c′.

To calculate the proportion of the total effect that is explained by the indirect pathway, we divided the indirect path estimate by the total effect, that is, (a×b)/(a×b+c′). This was complicated by instances where the direct and indirect effects counteracted one another—so-called suppressor effects—such that the total effect was less than the sum of the absolute effects. To overcome this, we used absolute values.^40^

The SEMs included linear and quadratic forms of age and pulse pressure, for completeness. In this case, the total mediation effect was reported as the sum of the absolute linear and quadratic pulse pressure mediation pathways: (a_1_×b)+(a_2_×b).

Model structure was based on theory and results of regression models. Model fit was assessed for the initial model using root mean square error of approximation and its CI, comparative fit index, and standardized root mean square residual. Good fit was defined as root mean square error of approximation <0.05, comparative fit index >0.97, and standardized root mean square residual <0.05.^41^ Statistical inferences on subsequent adaptations to model structure were made by comparing nested models via the likelihood ratio χ^2^ difference test (*P*<0.05). Model paths were considered significant if the bootstrapped (n=5000) CIs did not cross zero.^42^

SEM 1A explored whether the relationship between pulse pressure and processing speed (direct path, c′) was mediated by PSMD (indirect path, a×b), above the covariates of sex, handedness, and head motion (see Figure 1). Both linear and quadratic expressions of pulse pressure were used as mediators.

SEM 1A was next expanded to account for linear age in SEM 1B. The need to include linear age was assessed by comparing SEM 1B to a version in which the path between Speed and age was constrained to be equal to zero. Age was taken forwards only if it improved model fit. This process was repeated for the inclusion of quadratic age in SEM 1C.

The winning model version from SEM 1B to c informed the specification of an additional checking step, SEM 1D. This model investigated whether the mediation pathway was specific to pulse pressure, over and above other vascular signals. SEM 1D included linear and quadratic steady-state blood pressure and heart rate variability factors.

Next, we used multigroup SEM (SEM 2A–B) to investigate whether the mediation effects (in the winning model from the SEM 1 models) varied with age. It is possible that the strength of the entire mediation pathway changed across the lifespan in this broad sample (18–87 years of age). In our previous study,^12^ pulse pressure had unique effects on cognitive ability discrepancy (the difference between crystallized and fluid intelligence) only in older adults. Here, the cohort was categorized into 3 equally sized subgroups (n=190) of young (18–44 years), middle-aged (44–65 years), and old (65–87 years) adults. The structure of SEM 1A was repeated, now additionally using the lavaan argument for multiple subgroups. In SEM 2A, all model paths were constrained to be equal across age groups, whereas in SEM 2B, the a_1_ and a_2_ paths connecting pulse pressure linear and quadratic to PSMD, were allowed to vary across the 3 age groups. SEM 2A to b were then compared.

In the third set of models (SEM 3A–B), the mediation pathway was expanded to test whether the effects of pulse pressure on processing speed contribute to cognitive ability discrepancy, which is an approximation of longitudinal decline in fluid intelligence, as outlined previously.^12^ This tested whether individual differences in processing speed underpin downstream differences in fluid intelligence.^27^ The model expansion was also motivated by previous findings that the ability discrepancy was significantly associated with pulse pressure interacting with age.^12^ By including ability discrepancy, SEM 3A became a dual-mediation model (a_1_×b_1_×b_2_; see Figure 1). To check replication of Kievit et al,^27^ SEM 3B was also run on fluid intelligence alone, rather than ability discrepancy. In an attempt to move closer to understanding the causal sequence of events, the final model was compared with a version where the order of processing speed and fluid intelligence (or ability discrepancy) was reversed, that is, pulse pressure to PSMD to fluid intelligence to processing speed (but covariates were again unchanged).

## Results

### Participants

Characteristics of the 708 participants, aged 18 to 88 years, in the Cam-CAN Phase 2 are outlined in Supplemental Material, Participants and Supplementary Table S1, suggesting the cohort is cognitively healthy and free from dementia.

### Peak Width of Skeletonized Mean Diffusivity

PSMD values are illustrated in Figure S3. PSMD and linear pulse pressure correlated strongly and positively (*r*=0.43, *P*<0.001), with a relationship that appeared to be nonlinear (Figure S3). This motivated including quadratic vascular factors in analyses. Quadratic vascular effects are visualized in Figure S6.

### Structural Equation Models

**Figure 2.**
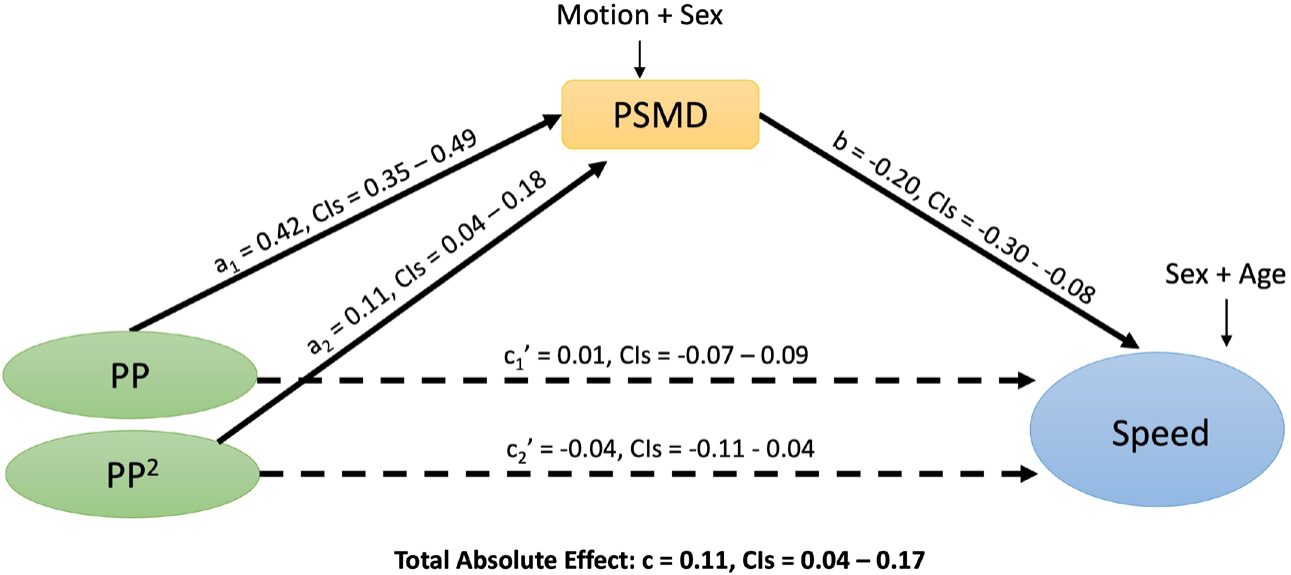
**Structural equation model 1B (n=570) with standardized betas.** Dashed lines represent insignificant results. Squares represent manifest variables and circles represent latent variables. CIs=95% bootstrapped CIs. PP indicates pulse pressure; and PSMD, peak width of skeletonized mean diffusivity.

The relationships between pulse pressure and PSMD, and between PSMD and processing speed, were significant in the linear regression models (Supplemental Section B: Results). This motivated exploring whether the relationship between pulse pressure and processing speed is mediated by PSMD. We investigated this using a series of structural equation models of increasing complexity (see Methods section: Analytical Integration of Vascular, White Matter and Cognitive Measures). SEM 1A modeled linear and quadratic pulse pressure contributions to processing speed, via PSMD, with a good fit (Table S12). In SEM 1A, the total absolute effect of linear and quadratic pulse pressure on processing speed was significant (*c*=0.41, CIs=[0.33–0.52]). This relationship was significantly mediated by PSMD (indirect absolute effect: [(a_1_×b)+(a_2_×b)]=0.29, CIs=[0.22–0.37]), accounting for 70% of the variance in the pulse pressure-speed relationship. This mediation was partial, meaning that the combined direct effects of linear and quadratic pulse pressure on speed remained significant (c_1_′+c_2_′=0.12, CIs=[0.04–0.25]). SEM 1B expanded the model to remove linear effects of age on processing speed (Figure 2; Table S13). The total absolute effect of linear and quadratic pulse pressure on processing speed remained significant (*c*=0.14, CIs=[0.09–0.28]), as did the mediation effect ([a_1_×b]+[a_2_×b])=0.10, CIs=[0.04–0.17]), accounting for 72% of the variance. This mediation was partial, in that the combined direct effects of linear and quadratic pulse pressure on speed remained significant (c_1_′+c_2_′=0.04, CIs=[0.01–0.16]). To assess the validity of including Age, SEM 1B was compared with a model where the path to Age was constrained to be equal to zero; the unconstrained model fit best (Table S14), showing the importance of adjusting for age.

**Figure 3.**
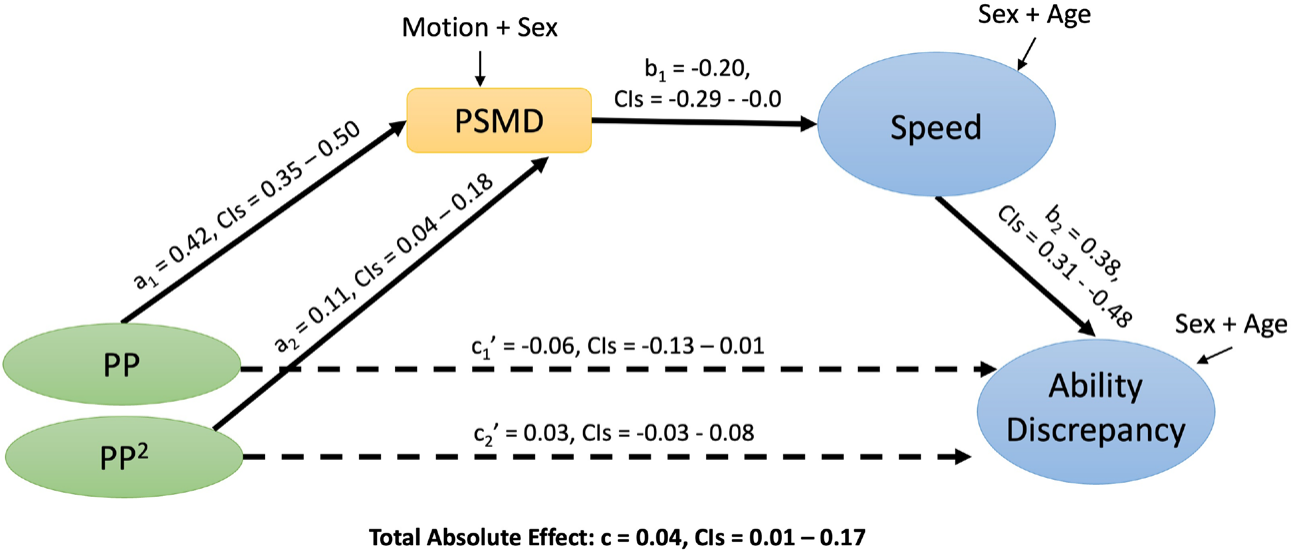
**Structural equation model 3B (n=564).** Shows standardized betas. Dashed lines represent insignificant results. Squares represent manifest variables and circles represent latent variables. PP indicates pulse pressure; and PSMD, peak width of skeletonized mean diffusivity.

SEM 1C included quadratic effects of age on processing speed (Table S15). The total absolute effect of linear and quadratic pulse pressures on processing speed was still significant (*c*=0.14, CIs=[0.08–0.27]), as was the mediation effect ([a_1_×b]+[a_2_×b])=0.09, CIs=[0.03–0.16]), accounting for 68% of the variance in the pulse pressure-speed relationship. SEM 1C was compared with a model where the path to Age^2^ was constrained, and the constrained model fit best (Table S16), consistent with regression analysis findings where Model 2B (excluding Age^2^) fit the data better than Model 2c (including Age^2^). Therefore Age^2^ was not taken forward into SEM 1D. In a specificity analysis, SEM 1D investigated whether the mediation pathway was specific to pulse pressure, over and above other vascular signals. SEM 1D included the steady-state blood pressure and heart rate variability (HRV) factors, in linear and quadratic forms (Figure S7; Table S17). The total absolute effect of all vascular factors on processing speed was significant (*c*=0.31, CIss=[0.24–0.62]). Pulse pressure to processing speed was significantly mediated by PSMD (indirect absolute effect: [(a_3_×b)+(a_4_×b)]=0.08, CIs=[0.03–0.13]), accounting for 24% of the variance in the pulse pressure-speed relationship. HRV to processing speed was also significantly mediated by PSMD (indirect absolute effect: [(a_5_×b)+(a_6_×b)]=0.07, CIs=[0.03–0.12]), accounting for 23% of the variance in the pulse pressure-speed relationship. The results observed in SEM 1A to 1d remained consistent when replacing the latent vascular estimates with observed measures of pulse pressure and steady-state blood pressure (Tables S18 through S23).It was possible that the strength of the entire mediation pathway changed with age, such that the effects of pulse pressure on white matter and cognition vary across the lifespan. To test this, we specified SEM 2A, where all model paths were constrained to be equal across 3 age groups, of young (18–44 years), middle (44–65 years), and old (65–87 years) adults. This model was compared with SEM 2B, where the paths connecting pulse pressure to PSMD were allowed to vary across the 3 age groups. SEM 2A fit the data best (Table S24), indicating that the strength of the mediation pathway is stable across the adult lifespan.The third set of models, motivated by the Watershed model, tested whether the effects of processing speed had downstream effects on higher cognition. SEM 3A tested whether the effects of pulse pressure on PSMD related to the ability discrepancy score, a proxy for longitudinal decline in fluid intelligence. To test whether the ability discrepancy was required, SEM 3A was compared with a version where the b_2_ path from processing speed to the ability discrepancy was constrained to zero, and the full model fit best (Table S25), suggesting that speed did relate to ability discrepancy. In SEM 3A (Table S26), the total absolute effect of pulse pressure on the ability discrepancy was significant (*c*=0.03, CIs=[0.02–0.04]). However, evidence for dual mediation of this relationship by PSMD and speed was borderline (indirect absolute effect: [(a_1_×b)+(a_2_×b)]=0.01, CIs=[0.00–0.02]). As the Watershed model was originally established using measures of fluid intelligence rather than discrepancy scores,^27^ we further examined whether the effects were significant when applied to fluid intelligence itself by testing SEM 3B. Again, we first confirmed that the b_2_ path from processing speed to the fluid intelligence in SEM 3B was needed, in that a model where it was constrained to zero was worse (Table S27). The total absolute effect of pulse pressure on fluid intelligence was significant (*c*=0.13, CIs=[0.06–0.21]) (Figure 3; Table S28), as was dual mediation of this relationship by PSMD and speed (indirect absolute effect: [(a_1_×b)+(a_2_×b)]=0.04, CIs=[0.01–0.07]), accounting for 31% of the variance in the pulse pressure-fluid intelligence relationship. The mediation was partial, in that the combined direct effects of linear and quadratic pulse pressure on fluid intelligence remained significant (c_1_′+c_2_″=0.09, CIs=[0.02–0.17]).

Finally, to get closer to causality, SEM 3B was compared with a model in which the direction of paths was reversed—such that fluid intelligence came before processing speed—using the watershed logic of Kievit et al.^27^ The expected order of variables fit best, independent of the inclusion or exclusion of covariates (Tables S29 and S30).

## Discussion

Using a multivariate approach, we confirmed the hypothesis that the effects of pulse pressure on cognition are at least partly explained by the integrity of white matter microstructure. There were 3 key findings. First, white matter microstructure significantly and substantially mediated the effect of pulse pressure on processing speed. Second, the strength of this mediation was stable across the adult lifespan. Third, the effects of pulse pressure on processing speed had downstream consequences for fluid intelligence.

These findings suggest that better management of pulse pressure, through addressing its vascular and hemodynamic contributors, may help reduce the risk of cognitive decline and dementia.^43^ We suggest that there is now sufficient evidence to motivate longitudinal and interventional studies to test whether better managing pulse pressure by targeting its vascular causes helps maintain white matter microstructure and preserve cognitive abilities in later life.

### PSMD and Processing Speed: A Sensitive Marker of Neurocognitive Aging

The PSMD values in Cam-CAN were similar to those in comparable population-based cohorts,^14,15^ and showed a similar profile of continuous increase with age, accelerating after 60 years of age.^14,15^ Here, the spread of PSMD values also increased substantially with age. This reinforces previous suggestions that PSMD may reflect individual differences in early brain aging.

Here, we replicated previous studies showed that increased PSMD is associated with reduced processing speed.^13,20–23^ Moreover, the association remained after adjusting for linear and quadratic effects of age, suggesting that white matter integrity is similarly important for processing speed across the adult lifespan. Processing speed has special status in studies of cognitive aging because it is thought to be the foundation for age-related differences in higher cognitive abilities.^24–26^ Our findings reinforce evidence that PSMD is a sensitive marker of neurocognitive aging, with additional potential relevance for dementia.

### Pulse Pressure and White Matter Microstructure

Pulse pressure correlated strongly and positively with PSMD, with a curvilinear relationship. The curve was relatively flat for low levels of pulse pressure, where incremental increases in pressure corresponded to only marginal increases in PSMD. The curve steepened almost exponentially at higher pressures. This could suggest that, while small fluctuations of normal levels in pulse pressure can be managed—for example, through vessel elasticity and the Windkessel effect—pressures beyond the limits of compensatory mechanisms penetrate the microcirculation, and drive cerebral damage with consequences for white matter integrity. Exponentially increasing damage could result from positive feedback loops driven by high pulse pressure, such as high pressure causing vessel calcification and remodeling, which in turn raises the pressure further.^2,43^ Alternatively, the exponential rise may reflect 2 distinct processes: a steady increase in systolic pressure from arterial stiffening and a biphasic trajectory in diastolic pressure—increasing during mid-life before gradually decreasing in later life^44–46^—together widening pulse pressure over time. In addition, blood pressure variability, which also increases with age and relates to both stiffness and pulse pressure,^47–49^ may further contribute by adding hemodynamic stress and accelerating damage.

In regression models, PSMD was uniquely associated with quadratic pulse pressure, over and above other vascular factors. A relationship between pulse pressure and white matter is consistent with previous studies on vascular factors and white matter hyperintensities.^8,10,50^ Pulse pressure may impair white matter microstructure by damaging and remodeling the microvasculature, leading to hypoperfusion and in turn diffuse axonal loss, demyelination, and inflammation.^2^

Independently of pulse pressure, the steady-state blood pressure factor was also related to PSMD, although not as strongly. It highlights the multivariate nature and complexity of vascular aging, as previously argued.^51^ Pulse pressure and steady-state blood pressure were related to PSMD, independent of medications, supporting the idea that pulse pressure affects white matter beyond antihypertensive medications.^9^ Here, while the linear model fit is best without medications, there were significant interactions. Pulse pressure interacted with beta-blockers and diuretics, while steady-state blood pressure interacted with antihypertensives. While some of these could be false positives, given the number of terms (tests) in these models, they should be explored in future work aimed at understanding the underlying mechanisms and developing interventions for cerebrovascular aging.

### Relating All 3: Pulse Pressure to White Matter to Cognition

The effects of pulse pressure on PSMD, and of PSMD on processing speed, were examined simultaneously in mediation models. In the first model, PSMD accounted for 70% of the direct relationship between pulse pressure and processing speed. This is in line with previous work associating pulse pressure with white matter microstructure,^8–11^ and white matter with cognitive performance.^13,16,20–23^ The mediation effect remained significant and substantial when covarying age, and the strength of the mediation was stable across groups of young, middle, and old-aged participants. The importance of this result is best considered in the context of the cohort in which it was studied: the biggest risk factor for increasing pulse pressure is age,^44,52^ and here, pulse pressure was studied in an adult cohort with a broad age range (18–88 years). For the mediation effect to be significant over and above age, in this cohort, suggests that there will be an even stronger effect when it is studied in a large sample with a smaller age range, such as in a group of middle-aged individuals in an interventional study.

A further analysis confirmed that pulse pressure affects white matter and cognition independently, over and above other vascular factors. However, there was additional significant and substantial mediation of HRV to processing speed. Low HRV has been linked to declines in processing speed in prior studies.^53–55^ This further highlights the multifactorial nature of vascular aging on brain health and cognitive function. Future work should explore whether pulse pressure and HRV contribute to loss of white matter integrity through similar or different mechanisms.

The model was next expanded to include the ability discrepancy, following the logic of the watershed hypothesis, namely that age-related declines in processing speed underpin downstream differences in higher cognition.^27^ Mediation of pulse pressure on the ability discrepancy was borderline significant. Given that the watershed hypothesis was originally established using measures of fluid intelligence,^27^ without adjusting for crystallized intelligence (as in the ability discrepancy score), we repeated this dual-mediation model with fluid intelligence as the outcome variable instead. Mediation of pulse pressure onto fluid intelligence, through PSMD and processing speed, was significant, even above age. The mediation pathway accounted for 31% of the total variance in fluid intelligence that was related to pulse pressure. Furthermore, reversing the paths (from fluid intelligence to pulse pressure) resulted in worse model fit. These results together support the watershed hypothesis,^27^ and further highlight the importance of pulse pressure in cognitive aging processes.

### Avenues to Treat Pulse Pressure

We speculate that better awareness and control of pulse pressure would slow the development and progression of white matter damage. Evidence from the regression models here suggests that β-blockers and diuretics interact with pulse pressure, changing its effect on white matter microstructure. Further work is needed to explore how different interventions—including lifestyle modifications, combination therapies, and pharmacological treatments—can mitigate these effects. In clinical practice, combining drugs can be more effective than doubling single-drug dosage and reduce side effects.^56^ For instance, vasodilators may reduce pulse pressure, and be combined with β-blockers to mitigate reflex tachycardia.^57^ Interventions that are currently being tested include: reducing arterial stiffness with aerobic exercise^58^; lowering systolic blood pressure with antihypertensives, to improve cardiovascular and cognitive health, in the Systolic Blood Pressure Intervention Trial (SPRINT) trial,^59–61^ and several multidomain interventions,^62^ including the Finnish Geriatric Intervention Study to Prevent Cognitive Impairment and Disability (FINGER) trial, which aims to lower systolic blood pressure to benefit cognition in older adults.^63,64^

### Strengths and Limitations

Cardiovascular, cerebral, and cognitive aging have often been studied in isolation. A key strength of this study is that we used an integrated, multivariate approach. To do this, we prioritized analyzing high-quality data. The Cam-CAN cohort offers an unusual combination of multiple vascular and brain measures with high-quality observations across multiple cognitive domains. This makes it an excellent test bed to develop new hypotheses about the multivariate effects of vascular aging. Developing ideas in this way is a necessary precursor to rigorous testing in complex and resource-demanding longitudinal and intervention studies. However, it should be kept in mind that inferences drawn from our current findings could be limited by the cross-sectional design of currently available Cam-CAN data, in which age effects are confounded by the effects of generational changes across people born in different years. When there is no temporal information, it is possible that the hypothesized direction—of x causes y via the mediator—is in fact reversed.^27^ Ideally, reverse causation would be theoretically implausible for any model developed in cross-sectional data. However, in our models, it is possible that instead of declines in cardiovascular health impairing cognitive abilities like processing speed, higher cognitive abilities in fact allow individuals to make lifestyle choices that improve their cardiovascular health. It is also possible that a unidirectional relationship in either direction is overly simplistic. For example, regarding blood pressure, it was thought that hypertension caused secondary endothelial damage. However, there is some evidence that endothelial dysfunction happens first, and further evidence that both converge into a reinforcing and bidirectional relationship.^65,66^ A similarly complex relationship may exist for pulse pressure and cognitive abilities, which would be difficult to untangle in cross-sectional data.

To try to address the possibility of reverse causation, we compared model fit with alternative orders of variables. The expected order—namely, pulse pressure to PSMD to processing speed—fit the data best. However, this comparison is not conclusive about the true causal direction, and future work should extend this with longitudinal and interventional study designs. It is also possible that age drives changes in pulse pressure, which in turn drive changes in white matter and cognition. In this case, age could be modeled on the left of the vascular-brain-cognition triangle. Investigating this in future work will require longitudinal cohorts.

The latent vascular factor which we refer to as pulse pressure here was created using factor analysis, as in our previous study,^12^ where it was primarily driven by the observed measures of pulse pressure, with a smaller, negative contribution from resting heart rate. Importantly, the sensitivity analysis revealed that the results remained consistent when observed measures of pulse pressure were used instead of the latent vascular measures, further supporting this terminology. While pulse pressure may only be a proxy for arterial stiffness, our approach of using a latent factor of pulse pressure may provide a more accurate representation of arterial stiffness. This is relevant given evidence that blood pressure estimates from the brachial artery—as in this study—can differ significantly from central aortic pressure.^67,68^ There is evidence that more direct measures, such as pulse wave velocity, explain greater variance in white matter hyperintensities.^69^ Latent variable modeling is widely used in medical^70,71^ and psychological^72,73^ research to improve construct validity when direct measurement is difficult. Currently, the Heart and Brain study is collecting multiple measures of pulse pressure, alongside ultrasound metrics of vessel stiffness, white matter lesions and cognitive abilities.^74^ The latent representation of these detailed multimodal measures of vascular pathology may contribute to better understanding the observed effects on brain and cognitive aging. However, interpreting these associations requires consideration of downstream mechanisms that may influence the brain’s vulnerability to pulsatile signals, independent of arterial stiffness itself. This includes myogenic contraction,^75^ endothelial dysfunction,^76^ and structural differences in intracranial arteries^76^—such as reduced media thickness and absence of the external elastic lamina—which may make the brain’s microcirculation particularly vulnerable to damage from high pulse pressure. Thus, future work should establish whether measuring only systolic and diastolic pressures is sufficient, or if a more comprehensive consideration of multiple vascular factors and modeling of their multifactorial nature, as proposed here, is required. This includes accounting for additional comorbidities such as hyperlipidemia, diabetes, and chronic kidney disease. This will be an important step in translating the findings into clinical practice, where systolic and diastolic pressures (and heart rate) are more routinely measured.

This study took an integrated approach to vascular, cerebral, and cognitive aging. However, the rate of biological aging may differ between organs within the same individual.^77^ Indeed, it is possible that individuals with high pulse pressure and accelerated vascular aging may have comparatively slower—and healthier—rates of cerebral aging, for other reasons. Future research should aim to better understand the nuances of cerebrovascular aging.

The calculation of global PSMD, as used here, sacrifices detail on regional specificity. This may be important in light of evidence that some brain regions are more vulnerable to pulsatile forces. Future work could investigate tract-specific estimates of PSMD or other derived diffusion metrics, such as microstructural complexity,^78^ and more conventional white matter hyperintensities that are routine in clinical magnetic resonance imaging,^79,80^ and their relation to measures of cerebrovascular health, including perfusion and cerebrovascular reactivity.^51,81,82^

### Conclusions

This study shows that white matter microstructure impairment mediates the effect of pulse pressure onto cognition. We propose that better managing pulse pressure by addressing vascular pathology—through lifestyle and therapeutic approaches—may help protect cerebral vessels and preserve cognitive abilities throughout life.

### Perspectives

We show that high pulse pressure—the difference between systolic and diastolic pressure—disrupts the microstructural integrity of white matter in the brain, leading to slower processing speed. The effects of pulse pressure were over and above other vascular factors. These findings suggest that pulse pressure is a distinct contributor to cognitive aging. Efforts to promote lifelong cognitive health could specifically target pulse pressure. An advantage of targeting pulse pressure is that it is relatively simple to assess with standard equipment available in routine clinical practice, or at home. Pulse pressure could be incorporated into the routine health screening for adults from middle age onwards. We propose that early monitoring would be most effective because vascular-mediated injury accumulates over a long time. Individuals with high pulse pressure could be guided by the evidence that it can be managed with lifestyle changes, exercise, nutrition and weight loss, and medications. New pharmaceutical interventions which specifically target pulse pressure are also now being developed. In general terms, there has been a lack of vascular therapeutic targets for dementia, due in part to limited understanding of the multivariate nature of vascular pathology. This is an area where research investment is overdue, and with broad potential benefits for global populations. Public health policies and clinical practice can be updated in the coming decade to target better managing pulse pressure to preserve cognition in old age. The first steps are to validate our findings in longitudinal cohorts and with complementary techniques.

## Article Information

### Acknowledgments

The authors thank the Cam-CAN (The Cambridge Center for Aging and Neuroscience) respondents and their primary care teams in Cambridge for their participation in this study, and colleagues at the MRC Cognition and Brain Sciences Unit Magnetoencephalogrphy (MEG) and Magnetic Resonance Imaging (MRI) facilities for their assistance. Further information about the Cam-CAN corporate authorship membership can be found at https://cam-can.mrc-cbu.cam.ac.uk/corpauth/#14 (list 14).

### Sources of Funding

Cam-CAN (The Cambridge Center for Aging and Neuroscience) was supported by the Biotechnology and Biological Sciences Research Council Grant BB/H008217/1. D.L.O. King was supported by a Doctoral Training program studentship awarded by the Biotechnology and Biological Sciences Research Council (BBSRC BB/M011194/1). K.A. Tsetanov was supported by Fellowship awards from the Guarantors of Brain (G101149) and Alzheimer’s Society, UK (grant number 602). R.N. Henson was supported by the UK Medical Research Council (SUAG/046 G101400) and the European Union’s Horizon 2020 research and innovation program (LifeBrain, Grant Agreement No. 732592). J.B. Rowe is funded by the Welcome Trust (220258), the Cambridge University Centre for Frontotemporal Dementia, the Medical Research Council (MC_UU_00030/14; MR/T033371/1); the National Institute for Health Research Cambridge Biomedical Research Centre (NIHR203312: BRC-1215-20014) and the Holt Fellowship. The views expressed are those of the authors and not necessarily those of the NIHR or the Department of Health and Social Care. For the purpose of open access, the author has applied a Creative Commons Attribution (CC BY) license to any Author Accepted Manuscript version arising from this submission.

### Disclosures

J.B. Rowe is a nonremunerated trustee of the Guarantors of Brain, Darwin College, and the PSP Association; he provides consultancy to Alzheimer Research UK, Astronautx, Asceneuron, Alector, Astex, Booster Therapeutics, ClinicalInk, CuraSen, CumulusNeuro, Eisai, Ferrer, SV Health, and Wave, and has research grants from AZ-Medimmune, Janssen, Lilly as industry partners in the Dementias Platform UK. The other authors report no conflicts.

### Supplemental Material

References 83–90

## Supplementary Material
