# Supplementary material for: Pulse Pressure Impairs Cognition via White Matter Disruption
Source: Hypertension. 2025 Jul 10;82(9):1480–91. doi: 10.1161/HYPERTENSIONAHA.124.24543 (PMC12366739; doi:10.1161/HYPERTENSIONAHA.124.24543)
Supplement: Supplementary file 1 [file hyp-82-1480-s001.docx]

Supplemental Material: Pulse Pressure Impairs Cognition via White Matter Disruption

Deborah L. O. King1,2, Richard N. Henson3,4,5, Marta Correia3,4, James B. Rowe1,4,5,6, Cam-CAN Consortium^4,5^ and Kamen A. Tsvetanov1,2

1. Department of Clinical Neurosciences, University of Cambridge, Cambridge CB2 0SP, United Kingdom.
2. Department of Psychology, University of Cambridge, Cambridge CB23 6HT, United Kingdom.
3. Department of Psychiatry, University of Cambridge CB2 2QQ, United Kingdom.
4. Medical Research Council Cognition and Brain Sciences Unit, Cambridge CB2 7EF, United Kingdom.
5. Cambridge Centre for Ageing and Neuroscience (Cam-CAN), University of Cambridge and MRC Cognition and Brain Sciences Unit, Cambridge CB2 7EF, United Kingdom.
6. Behavioural and Clinical Neuroscience Institute, Cambridge CB23 6HT, United Kingdom.

Short title: Pulse Pressure Impairs Cognition via White Matter

Corresponding author: Kamen A. Tsvetanov, Department of Clinical Neurosciences, Cambridge University, Cambridge CB2 0SZ, United Kingdom. Email: kat35@cam.ac.uk.

# Supplemental Material

## Supplemental Section A: Methods

### Participants

We studied participants in the population-based Cam-CAN cohort, which has deeply phentotyped data on approximately 700 adults, aged 18-88 years^32,33^. The methods were conducted in accordance with guidelines approved by Cambridgeshire 2 (now East of England—Cambridge Central) Research Ethics Committee (reference: 10/H0308/50), who approved all experimental protocols. All participants gave full, informed, written consent. Participants were recruited from Cambridge City GP surgeries, randomly to help maximise population representativeness of the cohort. The detailed recruitment pathway is outlined elsewhere^32^. Participants were cognitively healthy and free from a history of dementia, referral for dementia assessment or memory complaints, with Mini-Mental State Examination >24/30^83^, and revised Addenbrokes Cognitive Examination score >82/100. Education was reported across four categories of: none, GCSE or O-Level, A-Level, Degree (College or University).

### Cardiovascular Measures and Latent Vascular Factors

Observations were recorded for body mass index, heart rate, heart rate variability at low and high frequencies, and systolic and diastolic blood pressures. Medication status (binary on/off) was reported for drugs with cardiovascular relevance, across four categories: [1] anti-hypertensives; [2] beta blockers; [3] other diuretics; [4] dyslipidemics. These data were collected during Phase I of the study (2011-2013), when participants undertook a 2-hour in-home interview covering medication use, demographic, lifestyle, health and some cognitive data. Full details of these observations are available elsewhere^12,32^.

The vascular observations were processed following our previous methodology^12^, as summarised in Figure 1. In brief, systolic and diastolic blood pressure, and heart rate, were measured using the A&D Medical Digital Blood Pressure Monitor (UA-774). Measurements were taken from the participant’s dominant arm after being seated for at least 10 min and repeated 3 times in succession. The three repeated observations of systolic and diastolic blood pressures were used to calculate three iterations of steady state blood pressure (systolic + diastolic), and pulse pressure (systolic – diastolic). These three observations were condensed into a single latent variable, as were three observations of mean heart rate (pulse rate). The resulting variables were modelled, alongside body mass index and heart rate variability at low and high frequencies. These six vascular observations formed three latent vascular factors using Exploratory Factor Analysis. Steady state blood pressure loaded strongly onto the first latent factor, with a small contribution from body mass index. Pulse pressure loaded strongly onto the second latent factor, with a small negative contribution from mean heart rate. The measures of heart rate variability in both high and low frequencies loaded similarly onto the third latent factor. Each latent factor is referred to subsequently by the variable name with most prominent loading – namely, “steady-state blood pressure”, “pulse pressure” and “heart rate variability” – unless stated otherwise. The pulse pressure factor was the focus of the present theory-driven analysis, but to explore whether pulse pressure acts independently to other vascular signals, the factor scores for all three latent vascular factors were extracted and input to the statistical models outlined below. Sensitivity analysis examined whether the results remained consistent when using observed measures of pulse pressure and steady state blood pressure instead of latent vascular factors.

### Behavioural Tasks and Cognitive Measures

Processing speed was captured through response times in “simple” and “choice” tasks in a single latent factor of “speed”. In both tasks, the outcome measure was the time between presentation of a visual cue and pressing a button with a finger of the right hand (regardless of handedness). Handedness was assessed using the Edinburgh Handedness Inventory ^84^. In the simple task, there was only one type of cue, to which participants responded with their index finger only. In the choice task, different cues indicated which of four fingers to use. Full details are in ^32^. The mean and standard deviation of response times were calculated across the 40-60 trials with correct responses. Both reaction time tasks (in milliseconds) were positively skewed, therefore were log-transformed to better align with Gaussian distributions. The sign of the transformed scores was then flipped, such that higher scores represented faster responses, consistent with previous approaches ^85^. Finally, speed scores were standardised (Z-scored), and condensed into a single latent variable representing processing speed, using the “cfa” function in the lavaan package^38^. Missing data (simple task, N=1; choice task, N=1) were imputed using Full Information Maximum Likelihood, in cases where data were recoded for at least two observed variables, producing latent factor scores for n = 664. Factor score estimates for the latent variable were extracted for further analyses, below.

### Diffusion Tensor Imaging and White Matter Microstructure Measures

Pre-processing of the MRI data used the SPM12 software (Wellcome Department of Imaging Neuroscience; https://www.fil.ion.ucl.ac.uk/spm), release 4537, implemented in the Automatic Analysis pipeline, release 4.2^33,86^. In brief, 1mm isotropic T1- and T2-weighted images were bias-corrected for inhomogeneity of the magnetic field, segmented, and warped to match a gray matter template created from the whole CamCAN sample using SPM’s DARTEL toolbox. This template was subsequently affine transformed to standard MNI space. Details of the MR sequences are available here: https://camcan-archive.mrc-cbu.cam.ac.uk/dataaccess/pdfs/CAMCAN700_MR_params.pdf, and an XML summary of AA preprocessing is available here: https://camcan-archive.mrc-cbu.cam.ac.uk/dataaccess/ImagingScripts/mri_aa_release004_roistreams_v1_tasklist.xml.

The 2mm isotropic diffusion weighted imaging (DWI) were processed with a common pipeline that has been described in detail elsewhere^87^. In brief, DWI data were pre-processed including correction for noise and Gibbs ringing using DIPY tools (https://dipy.org/), eddy current distortions, and head movement using eddy in FSL (https://fsl.fmrib.ox.ac.uk/fsl/fslwiki/). After these pre-processing steps, six datasets were excluded from further analysis: two due to corrupted DWI, random variations in signal intensity resembling ‘salt and pepper’ noise^88^, and four due to excessive motion artefacts. Correction for B0 field inhomogeneities was not applied because reverse phase-encode direction data was not available. DTI fitting was performed by excluding the b=2000 s/mm^2^ data, using weighted linear least squares fitting in FSL, and mean diffusivity (MD) and fractional anisotropy (FA) maps were generated.

The fractional anisotropy (FA) and mean diffusivity (MD) maps were used to calculate the global peak width of skeletonised mean diffusivity (PSMD), following previous work^13^, shown in Figure S1. In brief, all participant’s FA maps were skeletonized, using the standard Tract-Based Spatial Statistics procedure, in FSL^89^. This created a group-specific mean FA template and a white matter skeleton. Each participant’s FA map was normalised to the group-specific FA template and projected onto the skeleton template (Figure S1, top row). The transformation and projection parameters were then applied to the participant’s MD map using tbss_non_FA script, creating a corresponding skeleton of MD values in group-specific space (Figure S1, bottom row). To avoid contamination of the MD skeleton by partial volume effects, particularly from cerebrospinal fluid, the MD skeleton was masked using the group-specific mean FA skeleton, thresholded above an FA value of 0.3. This tends to restrict voxels to cortical regions and exclude voxels close to ventricles, as advised^13^. In the resulting masked MD skeleton, the value of each voxel represents an MD value. A greater spread of MD values indicates global white matter breakdown. For each participant, the distribution of their MD values, across voxels within the masked skeleton, were plotted as a histogram (Figure S1). The difference between the 95th and 5th percentiles of the MD value was calculated (and divided by 1x10^6^ to convert to mm^2^/s, as in previous studies). A low PSMD value indicates greater uniformity in MD values throughout the white mater. A higher value indicates greater heterogeneity, assumed to reflect diffuse disruption across one or more white matter tracts. To understand how the calculated PSMD values corresponded to the input MD maps, we visualised the calculation process for three example participants (Figure S2).

DWI is sensitive to head motion, which creates artefacts that reduce diffusion estimates and tend to increase with age. To account for this, stripe index was used to estimate head motion effects and included as a covariate in subsequent analyses^78^.

### Linear Regression Models

In total, ten regression models tested two sets of nested hypotheses. The first set of models sought to understand the relationship between PSMD and pulse pressure in the presence of other variables (see equations below). Model 1a examined which latent vascular factors make unique contributions to PSMD, when accounting for sex, handedness and head motion. Model 1b investigated whether any relationships between latent vascular factors and PSMD held above linear age, and Model 1c investigated whether any relationships held above linear and quadratic age. Since latent vascular factors were highly correlated with age, any loss of significance in Models 1b and 1c could be due to age sharing variance with these factors. Models 1a-c were compared, and if age terms improved fit, they were taken into Models 1d-e. Model 1d included medications relevant to cardiovascular health, as chosen and reported previously^12^. Medication status was treated as a series of categorical variables. Model 1e included interactions between vascular factors and sex, because of evidence for sex-related differences in the relationship between pulse pressure and white matter microstructure^9^. If model comparisons showed that the inclusion of medications and sex, and interactions between vascular factors, did not improve overall fit in Models 1d-e, these would not be taken forward to the SEMs, below. The multiple linear regression models are outlined in Supplemental Section A.

The multiple linear regression models are outlined below using Wilkinson’s notation ^90^. In this model syntax, latent vascular factors expressing steady state blood pressure, pulse pressure and heart rate variability are abbreviated to SSBP, PP and HRV, respectively; “β” are the parameter estimates (coefficients); “ɛ” is the vector of residual errors; and interactions are indicated by “:”.

Model 1a:

PSMD ~ β_0_ + β_1_.SSBP + β_2_.SSBP^2^ + β_3_.PP + β_4_.PP^2^ + β_5_.HRV + β_6_.HRV^2^ + β_7_.Sex

+ β_8_.Head motion + β_9_.handedness + ɛ

Model 1b:

PSMD ~ β_0_ + β_1_.SSBP + β_2_.SSBP^2^ + β_3_.PP + β_4_.PP^2^ + β_5_.HRV + β_6_.HRV^2^ + β_7_.Sex

+ β_8_.Head motion + β_9_.handedness + β_10_.Age + ɛ

Model 1c:

PSMD ~ β_0_ + β_1_.SSBP + β_2_.SSBP^2^ + β_3_.PP + β_4_.PP^2^ + β_5_.HRV + β_6_.HRV^2^ + β_7_.Sex

+ β_8_.Head motion + β_9_.handedness + β_10_.Age + β_11_.Age^2^ + ɛ

Model 1d:
PSMD ~ β_0_ + β_1_.SSBP + β_2_.SSBP^2^ + β_3_.PP + β_4_.PP^2^ + β_5_.HRV + β_6_.HRV^2^
 + β_7_.Sex + β_8_.Head motion + β_9_.handedness +
 + β_10_.Age + β_11_.Age^2^
 + β_12_.Anti-Hypertensives + β_13_.Beta blockers + β_14_.Diuretics + β_15_.Dyslipidemics
 + β_16_.SSBP:Anti-Hypertensives + β_17_.SSBP^2^:Anti-Hypertensives

+ β_18_.PP:Anti-Hypertensives + β_19_.PP^2^:Anti-Hypertensives
 + β_20_.HRV:Anti-Hypertensives + β_21_.HRV^2^:Anti-Hypertensives
 + β_22_.SSBP:Beta blockers + β_23_.SSBP^2^:Beta blockers

+ β_24_.PP:Beta blockers + β_25_.PP^2^:Beta blockers
 + β_26_.HRV:Beta blockers+ β_27_.HRV^2^:Beta blockers
 + β_28_.SSBP:Diuretics + β_29_.SSBP^2^:Diuretics

+ β_30_.PP:Diuretics + β_31_.PP^2^:Diuretics

+ β_32_.HRV:Diuretics+ β_33_.HRV^2^:Diuretics
 + β_34_.SSBP:Dyslipidemics + β_35_.SSBP^2^:Dyslipidemics

+ β_36_.PP:Dyslipidemics + β_37_.PP^2^:Dyslipidemics
 + β_38_.HRV:Dyslipidemics + β_39_.HRV^2^:Dyslipidemics

+ ɛ

Model 1e:

PSMD ~ β_0_ + β_1_.SSBP + β_2_.SSBP^2^ + β_3_.PP + β_4_.PP^2^ + β_5_.HRV + β_6_.HRV^2^ + β_7_.Sex

+ β_8_.Head motion + β_9_.handedness + β_10_.Age + β_11_.Age^2^ + β_12_.SSBP:Sex

+ β_13_.SSBP^2^:Sex + β_14_.PP:Sex + β_15_.PP^2^:Sex + β_16_.HRV:Sex + β_17_.HRV^2^:Sex + ɛ

Regression models 2a-d attempted to replicate previous findings that PSMD related to processing speed ^13,21–24^. Model 2a accounted for sex, handedness and head motion. Model 2b additionally tested whether the effect of PSMD on speed held when accounting for linear age. Model 2c additionally accounted for quadratic age. If model comparisons showed additional terms did not improve model fit, these terms would not be taken into subsequent SEMs, below.

Model 2a:

Processing Speed ~ β_0_ + β_1_.PSMD + β_2_.Sex + β_3_.handedness + ɛ

Model 2b:

Processing Speed ~ β_0_ + β_1_.PSMD + β_2_.Sex + β_3_.handedness + β_4_.Age + ɛ

Model 2c:

Processing Speed ~ β_0_ + β_1_.PSMD + β_2._Sex + β_3_.handedness + β_4_.Age + β_5_.Age^2^ + ɛ

## Supplemental Section B: Results

Vascular observations were condensed into three latent vascular factors, using exploratory factor analysis, as in our previous study ^12^. The latent factors predominantly expressed steady state blood pressure, pulse pressure and heart rate variability. All latent vascular factors correlated significantly with age (Figure S3).

The four RT observations (Figure S4) were condensed into a latent variable representing processing speed (Figure S5). Processing speed and fluid intelligence both showed strong associations with age, and correlated significantly with the three latent vascular factors, with substantial effect sizes (Figure S3)

### Peak Width of Skeletonized Mean Diffusivity

PSMD was calculated for 620 participants. The values of PSMD are illustrated for three example participants in Figure S2. PSMD increased strongly with age (r = 0.75, p <0.001), with an uptick after 60 years of age (Figure S3). Head motion also increased with age (r = 0.16, p <0.001), and PSMD correlated moderately with head motion (r = 0.17, p <0.001). However, the correlation between PSMD and age remained strong after accounting for head motion (r = 0.75, p<0.001). Head motion was a covariate on PSMD in all subsequent analyses.

Correlations for complete case data across multimodal measures (n=564) are shown in Figure S3.

### Multiple Linear Regression

In the regression models predicting PSMD, Model 1a (Table S2) showed a significant positive association from both linear pulse pressure (β = 0.25, p <0.001), and quadratic pulse pressure (β = 0.12, p <0.001). This was consistent with a relationship between pulse pressure and PSMD that was stronger at higher values of pulse pressure. There was a significant negative relationship with linear HRV (β = -0.33, p<0.001). PSMD also showed significant positive relationships with sex (β = 0.21, p<0.001) and head motion (β = 0.13, p<0.001).

The model was next expanded in three stages, first to include linear Age in Model 1b (Table S3). This improved model fit (Table S4), therefore linear Age was taken forwards into Model 1c, which additionally included quadratic Age (Table S5). This significantly improved model fit further, therefore linear and quadratic Age were taken into subsequent models. In Model 1d (Table S6), there were significant interactions between pulse pressure and both betablockers and diuretics, and between steady state blood pressure and anti-hypertensives. However, there were no improvements across fit indices when including medications in Model 1d, or interactions with sex in Model 1e (Table S7). Overall, Model 1c fit best.

In Model 1c (Table S5), PSMD was significantly and positively associated with quadratic pulse pressure, over and above other vascular factors and linear and quadratic effects of Age (β = 0.05, p = 0.02). Head motion and sex also continued to show age-independent effects. Given these significant results, head motion, sex and both linear and quadratic effects of pulse pressure were taken into the pre-planned SEMs, below. There was also a significant effect of linear steady-state blood pressure (β = 0.05, p = 0.04); this was explored further in the post-hoc SEM, which included all latent vascular factors.

Taken together, the results of the regression Models 1a-e motivated including linear and quadratic expansions of pulse pressure in the subsequent SEM analyses. Additionally, since both linear and quadratic effects of Age explained significant variance in PSMD, above pulse pressure (in Model 1c), both age terms were also incorporated into the SEM analyses.

In the regression models predicting Processing Speed, Model 2a (Table S8) showed a significant negative relationship with PSMD (β = -0.62, p <0.001), and a positive relationship with sex (β = 0.29, p <0.001). These effects held above linear Age in Model 2b (Table S9), which improved model fit (Table S11), Model 2c with quadratic age fit worse (Table S10). Overall, Model 2b fit best. Importantly, the effect of PSMD on Processing Speed remained significant over and above linear (Model 2b) and quadratic (Model 2c) effects of Age.

## Supplemental Reference Section

## Supplemental Tables

Table S1. Demographic information for 708 participants, by equally split age groups. One decimal place is reported where data are continuous. GCSE – The General Certificate of Secondary Education, MMSE – Mini Mental State Examination; SD – standard deviation

|  |  | Young | Middle | Old |
| --- | --- | --- | --- | --- |
| n |  |  |  |  |
|  |  | 164 | 325 | 219 |
|  |  |  |  |  |
| Age (years) | |  |  |  |
|  | range | 18-37 | 38-67 | 68-99 |
|  | mean (SD) | 29.53 (5.49) | 52.51 (8.51) | 76.61 (5.41) |
|  |  |  |  |  |
| Sex |  |  |  |  |
|  | female | 85 (51.8) | 166 (51.1) | 108 (49.3) |
|  |  |  |  |  |
| MMSE (max 30) | |  |  |  |
|  | mean (SD) | 29.22 (1.28) | 29.00 (1.19) | 28.17 (1.53) |
|  |  |  |  |  |
| Education | |  |  |  |
|  | No qualifications tried, n (%) | 1 (0.6) | 10 (3.1) | 35 (16.0) |
|  | GCSE/O-levels, n (%) | 18 (11.0) | 46 (14.2) | 32 (14.6) |
|  | A-levels, n (%) | 25 (15.2) | 60 (18.5) | 53 (24.2) |
|  | Degree, n (%) | 119 (72.6) | 209 (64.3) | 98 (44.7) |
|  |  |  |  |  |
| BMI (kg/m2) | |  |  |  |
|  | mean (SD) | 23.79 (3.97) | 25.94 (4.90) | 27.11 (4.01) |
|  |  |  |  |  |
| Systolic (mmHg), mean (SD) | |  |  |  |
|  | mean (SD) | 112.25 (11.03) | 118.78 (14.63) | 130.30 (19.41) |
|  |  |  |  |  |
| Diastolic (mmHg) | |  |  |  |
|  | mean (SD) | 69.92 (8.66) | 74.97 (10.40) | 72.58 (10.77) |
|  |  |  |  |  |
| Heart Rate (beats/minute) | |  |  |  |
|  | mean (SD) | 65.45 (10.05) | 64.50 (9.47) | 67.97 (11.90) |
|  |  |  |  |  |
| Medications | |  |  |  |
|  | Any, n(%) | 0 (0.0) | 26 (8.0) | 82 (37.4) |
|  | Anti-hypertensives, n(%) | 0 (0.0) | 26 (8.0) | 82 (37.4) |
|  | Beta Blockers, n(%) | 0 (0.0) | 3 (0.9) | 21 (9.6) |
|  | Other diuretics, n(%) | 0 (0.0) | 9 (2.8) | 29 (13.2) |
|  | Dyslipidemics, n(%) | 0 (0.0) | 28 (8.6) | 62 (28.3) |
|  |  |  |  |  |
| Hypertension | |  |  |  |
|  | n(%) | 8 (4.9) | 38 (11.7) | 93 (42.5) |
|  |  |  |  |  |
| Hyperlipidaemia | |  |  |  |
|  | n (%) | 0 (0.0) | 46 (14.2) | 50 (22.8) |
|  |  |  |  |  |
| Diabetes | |  |  |  |
|  | n(%) | 0 (0.0) | 9 (2.8) | 21 (9.6) |
|  |  |  |  |  |
| General Health | |  |  |  |
|  | Excellent, n(%) | 31 (18.9) | 113 (34.8) | 62 (28.3) |
|  | Good, n(%) | 102 (62.2) | 157 (48.3) | 135 (61.6) |
|  | Fair, n(%) | 26 (15.9) | 48 (14.8) | 21 (9.6) |
|  | Poor, n(%) | 4 (2.4) | 7 (2.2) | 0 (0.0) |

Table S2. Results of Model 1a (n = 611, DoF = 602 residual standard error = 0.60) with dependent variable PSMD. Significant effects (p<0.05) are shown in bold.

| Predictors | Standard β | Standard Error | Confidence Intervals | P |
| --- | --- | --- | --- | --- |
| SSBP | 0.01 | 0.03 | -0.06 – 0.07 | 0.817 |
| SSBP^2^ | 0.00 | 0.03 | -0.05 – 0.06 | 0.941 |
| PP | 0.25 | 0.03 | 0.19 – 0.31 | **<0.001** |
| PP^2^ | 0.12 | 0.03 | 0.07 – 0.18 | **<0.001** |
| HRV | -0.33 | 0.03 | -0.38 – -0.27 | **<0.001** |
| HRV^2^ | 0.03 | 0.03 | -0.03 – 0.08 | 0.314 |
| Head motion | 0.13 | 0.03 | 0.07 – 0.18 | **<0.001** |
| Sex | 0.21 | 0.06 | 0.10 – 0.32 | **<0.001** |
| Handedness | 0.03 | 0.03 | -0.03 – 0.08 | 0.354 |

Table S3. Results of Regression Model 1b (n = 611, DoF = 601, residual standard error = 0.49) with dependent variable PSMD. Significant effects (p<0.05) are shown in bold.

| Predictors | Standard β | Standard Error | Confidence Intervals | p |
| --- | --- | --- | --- | --- |
| SSBP | 0.00 | 0.02 | -0.05 – 0.05 | 0.908 |
| SSBP^2^ | 0.04 | 0.02 | 0.00 – 0.09 | **0.043** |
| PP | 0.07 | 0.03 | 0.02 – 0.12 | **0.008** |
| PP^2^ | 0.07 | 0.02 | 0.03 – 0.11 | **0.002** |
| HRV | -0.01 | 0.03 | -0.06 – 0.04 | 0.653 |
| HRV^2^ | 0.04 | 0.02 | -0.00 – 0.08 | 0.072 |
| Head motion | 0.06 | 0.02 | 0.02 – 0.10 | **0.003** |
| Sex | 0.26 | 0.04 | 0.18 – 0.34 | **<0.001** |
| Handedness | 0.01 | 0.02 | -0.03 – 0.05 | 0.665 |
| Age | 0.61 | 0.03 | 0.56 – 0.67 | **<0.001** |

Table S4. Results of comparisons on Models 1a to 1d, using AIC and BIC, and the sum of squares derived from ANOVA comparisons.

|  | Difference in AIC | Difference in BIC | Difference in Sum  Of Squares |
| --- | --- | --- | --- |
| Model 1a vs 1b | 297.45 | 293.03 | 158.60 |
| Model 1b vs 1c | 73.12 | 68.71 | 29.13 |
| Model 1c vs 1d | 1.63 | -121.99 | 19.96 |
| Model 1c vs 1e | -8.88 | -35.37 | 1.13 |

Table S5. Results of Regression Model 1c (n = 611, DoF = 600, residual standard error = 0.46) with dependent variable PSMD. Significant effects (p<0.05) are shown in bold.

| Predictors | Standard β | Standard Error | Confidence Intervals | p |
| --- | --- | --- | --- | --- |
| SSBP | 0.05 | 0.02 | 0.00 – 0.10 | **0.043** |
| SSBP^2^ | 0.04 | 0.02 | -0.00 – 0.08 | 0.072 |
| PP | 0.00 | 0.03 | -0.05 – 0.05 | 0.871 |
| PP^2^ | 0.05 | 0.02 | 0.01 – 0.09 | **0.025** |
| HRV | 0.02 | 0.02 | -0.05 – 0.05 | 0.521 |
| HRV^2^ | 0.02 | 0.02 | -0.02 – 0.06 | 0.329 |
| Head motion | 0.04 | 0.02 | 0.00 – 0.08 | **0.043** |
| Sex | 0.28 | 0.04 | 0.20 – 0.36 | **<0.001** |
| Handedness | 0.01 | 0.02 | -0.03 – 0.04 | 0.735 |
| Age | 0.68 | 0.03 | 0.63 – 0.73 | **<0.001** |
| Age^2^ | 0.19 | 0.02 | 0.15 – 0.23 | **<0.001** |

Table S6. Results of Regression Model 1d (n = 611, DoF = 572, residual standard error = 0.42) with dependent variable PSMD. Significant effects (p<0.05) are shown in bold. The adjusted p-value with Bonferroni corrections for this non-winning model, containing 38 orthogonal tests, would be p<0.0013.

| Predictors | Standard β | Standard Error | Confidence Intervals | p |
| --- | --- | --- | --- | --- |
| SSBP | 0.07 | 0.03 | 0.02 – 0.12 | **0.005** |
| SSBP^2^ | 0.06 | 0.02 | 0.02 – 0.10 | **0.007** |
| PP | 0.01 | 0.03 | -0.04 – 0.07 | 0.692 |
| PP^2^ | 0.06 | 0.02 | 0.01 – 0.10 | **0.021** |
| HRV | 0.03 | 0.03 | -0.03 – 0.08 | 0.340 |
| HRV^2^ | 0.02 | 0.02 | -0.02 – 0.07 | 0.313 |
| Head motion | 0.02 | 0.02 | -0.03 – 0.07 | 0.400 |
| Sex | 0.25 | 0.04 | 0.17 – 0.33 | **<0.001** |
| Handedness | 0.01 | 0.02 | -0.03 – 0.04 | 0.709 |
| Age | 0.64 | 0.03 | 0.59 – 0.70 | **<0.001** |
| Age^2^ | 0.17 | 0.02 | 0.13 – 0.21 | **<0.001** |
| Anti-Hypertensives | 0.22 | 0.12 | -0.02 – 0.47 | 0.072 |
| Beta Blockers | -0.10 | 0.42 | -0.93 – 0.72 | 0.801 |
| Diuretics | 0.35 | 0.27 | -0.17 – 0.88 | 0.192 |
| Dyslipidemics | 0.23 | 0.12 | -0.00 – 0.45 | 0.052 |
| SSBP : Anti-Hypertensives | -0.28 | 0.11 | -0.50 – -0.06 | **0.012** |
| SSBP^2^ : Anti-Hypertensives | 0.00 | 0.12 | -0.24 – 0.23 | 0.998 |
| SSBP : Betablockers | 0.13 | 0.25 | -0.36 – 0.61 | 0.603 |
| SSBP^2^ : Betablockers | 0.15 | 0.41 | -0.66 – 0.96 | 0.714 |
| SSBP : Diuretics | 0.20 | 0.15 | -0.10 – 0.51 | 0.186 |
| SSBP^2^ : Diuretics | -0.16 | 0.16 | -0.47 – 0.15 | 0.319 |
| SSBP : Statins | -0.04 | 0.11 | -0.26 – 0.19 | 0.757 |
| SSBP^2^ : Statins | 0.00 | 0.13 | -0.26 – 0.25 | 0.977 |
| PP : Anti-Hypertensives | -0.02 | 0.10 | -0.21 – 0.17 | 0.859 |
| PP^2^ : Anti-Hypertensives | 0.01 | 0.08 | -0.15 – 0.17 | 0.937 |
| PP : Betablockers | 0.91 | 0.32 | 0.27 – 1.55 | **0.005** |
| PP^2^ : Betablockers | -0.85 | 0.37 | -1.58 – -0.13 | **0.023** |
| PP : Diuretics | -0.46 | 0.19 | -0.84 – -0.08 | **0.018** |
| PP^2^ : Diuretics | 0.80 | 0.26 | 0.29 – 1.31 | **0.002** |
| PP : Statins | -0.11 | 0.08 | -0.26 – 0.04 | 0.139 |
| PP^2^ : Statins | -0.03 | 0.06 | -0.15 – 0.09 | 0.602 |
| HRV : Anti-Hypertensives | -0.20 | 0.12 | -0.44 – 0.04 | 0.098 |
| HRV^2^ : Anti-Hypertensives | -0.13 | 0.10 | -0.33 – 0.07 | 0.189 |
| HRV : Betablockers | 0.22 | 0.36 | -0.48 – 0.92 | 0.544 |
| HRV^2^ : Betablockers | 0.11 | 0.35 | -0.57 – 0.80 | 0.743 |
| HRV : Diuretics | 0.28 | 0.32 | -0.36 – 0.92 | 0.389 |
| HRV^2^ : Diuretics | 0.18 | 0.24 | -0.29 – 0.65 | 0.451 |
| HRV : Statins | -0.01 | 0.13 | -0.26 – 0.24 | 0.924 |
| HRV^2^ : Statins | -0.03 | 0.07 | -0.17 – 0.11 | 0.685 |

Table S7. Results of Regression Model 1e (n = 611, DoF = 594, residual standard error = 0.44) with dependent variable Processing Speed. Significant effects (p<0.05) are shown in bold.

| Predictors | Standard β | Standard Error | Confidence Intervals | p |
| --- | --- | --- | --- | --- |
| SSBP | 0.07 | 0.03 | -0.00 – 0.13 | 0.052 |
| SSBP^2^ | 0.04 | 0.03 | -0.01 – 0.09 | 0.154 |
| PP | -0.04 | 0.04 | -0.11 – 0.03 | 0.227 |
| PP^2^ | 0.06 | 0.03 | 0.00 – 0.11 | **0.045** |
| HRV | 0.02 | 0.03 | -0.04 – 0.09 | 0.425 |
| HRV^2^ | 0.03 | 0.02 | -0.02 – 0.08 | 0.239 |
| Head motion | 0.04 | 0.02 | 0.00 – 0.08 | **0.037** |
| Sex | 0.28 | 0.04 | 0.21 – 0.36 | **<0.001** |
| Handedness | 0.00 | 0.02 | -0.03 – 0.04 | 0.795 |
| Age | 0.68 | 0.03 | 0.63 – 0.74 | **<0.001** |
| Age^2^ | 0.19 | 0.02 | 0.15 – 0.23 | **<0.001** |
| SSBP:Sex | -0.02 | 0.05 | -0.12 – 0.07 | 0.637 |
| SSBP^2^:Sex | 0.00 | 0.04 | -0.08 – 0.08 | 0.946 |
| PP:Sex | 0.08 | 0.05 | -0.01 – 0.18 | 0.075 |
| PP^2^:Sex | -0.05 | 0.04 | -0.14 – 0.03 | 0.206 |
| HRV:Sex | -0.02 | 0.04 | -0.10 – 0.06 | 0.665 |
| HRV^2^:Sex | -0.02 | 0.04 | -0.10 – 0.06 | 0.652 |

Table S8. Results of Regression Model 2a (n = 579, DoF = 576, residual standard error = 0.73) with dependent variable Processing Speed. Significant effects (p<0.05) are shown in bold.

| Predictors | Standard β | Standard Error | Confidence Intervals | P |
| --- | --- | --- | --- | --- |
| PSMD | -0.62 | 0.03 | -0.69 – -0.55 | <0.001 |
| Sex | 0.29 | 0.07 | 0.16 – 0.42 | <0.001 |
| Handedness | 0.02 | 0.03 | -0.05 – 0.09 | 0.648 |

Table S9. Results of Regression Model 2b (n = 579, DoF = 575, residual standard error = 0.66) with dependent variable Processing Speed. Significant effects (p<0.05) are shown in bold.

| Predictors | Standard β | Standard Error | Confidence Intervals | P |
| --- | --- | --- | --- | --- |
| PSMD | -0.24 | 0.05 | -0.33 – -0.15 | <0.001 |
| Sex | 0.18 | 0.06 | 0.06 – 0.30 | <0.01 |
| Handedness | 0.03 | 0.03 | -0.02 – 0.09 | 0.243 |
| Age | -0.49 | 0.04 | -0.58 – -0.40 | <0.001 |

Table S10. Results of Regression Model 2c (n = 579, DoF = 574, residual standard error = 0.66). Significant effects (p<0.05) are shown in bold.

| Predictors | Standard β | Standard Error | Confidence Intervals | P |
| --- | --- | --- | --- | --- |
| PSMD | -0.22 | 0.05 | -0.32 – -0.13 | **<0.001** |
| Sex | 0.17 | 0.06 | 0.05 – 0.29 | **<0.01** |
| Handedness | 0.03 | 0.03 | -0.02 – 0.09 | 0.243 |
| Age | -0.5 | 0.05 | -0.60 – -0.41 | **<0.001** |
| Age^2^ | -0.03 | 0.03 | -0.09 – 0.03 | 0.385 |

Table S11. Results of comparisons on Models 2a to 2c, using AIC and BIC, and the sum of squares derived from ANOVA comparisons.

|  | Difference in AIC | Difference in BIC | Difference in Sum  Of Squares |
| --- | --- | --- | --- |
| Model 2a vs 2b | 97.49 | 93.13 | 58.66 |
| Model 2b vs 2c | -1.41 | -5.78 | 0.32 |

Table S12. Structural Equation Model 1A. Significant effects where confidence intervals do not cross zero are in bold.

| Dependent variable | Predictor variable | Label | Standard β | Standard Error | z | p | Confidence Intervals |
| --- | --- | --- | --- | --- | --- | --- | --- |
| Speed | PP | c_1_’ | -0.10 | 0.04 | -2.25 | **0.02** | -0.18 – -0.01 |
| Speed | PP^2^ | c_2_^’^ | -0.03 | 0.04 | -0.73 | 0.47 | -0.11 – 0.05 |
| PSMD | PP | a_1_ | 0.42 | 0.04 | 11.15 | **<0.01** | 0.35 – 0.49 |
| PSMD | PP^2^ | a_2_ | 0.11 | 0.04 | 3.07 | **<0.01** | 0.04 – 0.18 |
| Speed | PSMD | b_1_ | -0.54 | 0.04 | -12.53 | **<0.01** | -0.63 - -0.46 |
| PSMD | Sex |  | 0.10 | 0.08 | 2.54 | **0.01** | 0.04 – 0.35 |
| PSMD | Head Motion |  | 0.16 | 0.04 | 3.68 | **<0.01** | 0.10 – 0.27 |
| Speed | Sex |  | 0.13 | 0.07 | 3.66 | **<0.01** | 0.12 – 0.41 |
| TAM of  PP + PP^2^ |  | (a_1_ x b) +  (a_2_ x b) | 0.29 | 0.04 | 7.3 | **<0.01** | 0.22 – 0.37 |

Abbreviations: PP, pulse pressure; PSMD, peak width of skeletonized mean diffusivity; TAM – Total Absolute Mediation.

Table S13. Structural Equation Model 1B. Significant effects where confidence intervals do not cross zero are in bold.

| Dependent variable | Predictor variable | Label | Standard β | Standard Error | z | p | Confidence Intervals |
| --- | --- | --- | --- | --- | --- | --- | --- |
| Speed | PP | c_1’_ | 0.01 | 0.04 | 0.15 | 0.89 | -0.07 – 0.09 |
| Speed | PP^2^ | c_2’_ | -0.04 | 0.04 | -0.92 | 0.36 | -0.11 – 0.04 |
| PSMD | PP | a_1_ | 0.42 | 0.04 | 11.15 | **<0.01** | 0.35 – 0.49 |
| PSMD | PP^2^ | a_2_ | 0.11 | 0.04 | 3.07 | **<0.01** | 0.04 – 0.18 |
| Speed | PSMD | b_1_ | -0.20 | 0.06 | -3.40 | **<0.01** | -0.30 – -0.08 |
| PSMD | Sex |  | 0.10 | 0.08 | 2.54 | **0.01** | 0.04 – 0.35 |
| PSMD | Head Motion |  | 0.16 | 0.04 | 3.68 | **<0.01** | 0.10 – 0.27 |
| Speed | Sex |  | 0.13 | 0.07 | 1.93 | **0.05** | -0.00 – 0.26 |
| Speed | Age |  | -0.54 | 0.05 | -10.00 | **<0.01** | -0.61 – -0.41 |
| TAM of  PP + PP^2^ |  | (a_1_ x b) +  (a_2_ x b) | 0.11 | 0.03 | 3.05 | **<0.01** | 0.04 – 0.17 |

Abbreviations: PP, pulse pressure; PSMD, peak width of skeletonized mean diffusivity; TAM – Total Absolute Mediation.

Table S14. Likelihood ratio test results on Structural Equation Model 1B, comparing the full model to a version where the path to Age was essentially removed, by constraining it to be equal to zero.

| Model | DF | AIC | BIC | Chi-sq | p |
| --- | --- | --- | --- | --- | --- |
| SEM 1b, full model | 2 | 9, 889 | 10, 002 | 371.96 |  |
| SEM 1b, constraining Age | 3 | 9, 982 | 10, 091 | 467.14 | <0.001 |

Abbreviations: AIC, Akiake Information Criterion; BIC, Bayesian Information Criterion; DF, degrees of freedom.

Table S15. Structural Equation Model 1C. Significant effects where confidence intervals do not cross zero are in bold.

| Dependent variable | Predictor  variable | label | Standard β | Standard Error | z | p | Confidence  intervals |
| --- | --- | --- | --- | --- | --- | --- | --- |
| Speed | PP | c_1’_ | 0.01 | 0.04 | 0.30 | 0.77 | -0.07 – 0.10 |
| Speed | PP^2^ | c_2’_ | -0.03 | 0.04 | -0.89 | 0.38 | -0.11 – 0.04 |
| PSMD | PP | a_1_ | 0.42 | 0.04 | 11.15 | **<0.01** | 0.35 – 0.49 |
| PSMD | PP^2^ | a_2_ | 0.11 | 0.04 | 3.07 | **<0.01** | 0.04 – 0.18 |
| Speed | PSMD | b_1_ | -0.19 | 0.06 | -3.00 | **<0.01** | -0.29 – -0.06 |
| PSMD | Sex |  | 0.10 | 0.08 | 2.54 | **0.01** | 0.04 – 0.35 |
| PSMD | Head Motion |  | 0.16 | 0.04 | 3.68 | **<0.01** | 0.10 – 0.27 |
| Speed | Sex |  | 0.07 | 0.07 | 1.74 | 0.08 | -0.01 – 0.26 |
| Speed | Age |  | -0.55 | 0.06 | -9.50 | **<0.01** | -0.63 – -0.42 |
| Speed | Age^2^ |  | -0.03 | 0.03 | -0.80 | 0.42 | -0.09 – 0.04 |
| TAM of PP + PP^2^ |  | (a_1_ x b) +  (a_2_ x b) | 0.10 | 0.03 | 2.73 | **0.01** | 0.03 – 0.16 |

Abbreviations: PP, pulse pressure; PSMD, peak width of skeletonized mean diffusivity; TAM – Total Absolute Mediation.

Table S16. Likelihood ratio test results comparing Structural Equation Model 1C, comparing the full model to a version where the path to Age^2^ was essentially removed, by constraining it to be equal to zero.

| Model | DF | AIC | BIC | Chi-sq | p |
| --- | --- | --- | --- | --- | --- |
| SEM 1c, full model | 3 | 11, 471 | 11, 615 | 436.01 |  |
| SEM 1c, constraining Age^2^ | 4 | 11, 470 | 11, 609 | 436.62 | 0.43 |

Abbreviations: AIC, Akiake Information Criterion; BIC, Bayesian Information Criterion; DF, degrees of freedom.

Table S17. Structural Equation Model 1D. Significant effects where confidence intervals do not cross zero are in bold.

| Dependent variable | Predictor  variable | label | Standard β | Standard Error | z | p | Confidence Intervals |
| --- | --- | --- | --- | --- | --- | --- | --- |
| Speed | SSBP | c_1’_ | 0.03 | 0.04 | 0.73 | 0.47 | -0.05 – 0.10 |
| Speed | SSBPP^2^ | c_2’_ | 0.01 | 0.03 | 0.40 | 0.69 | -0.05 – 0.08 |
| Speed | PP | c_3’_ | 0.00 | 0.04 | -0.07 | 0.95 | -0.09 – 0.08 |
| Speed | PP^2^ | c_4’_ | -0.04 | 0.04 | -1.01 | 0.31 | -0.12 – 0.03 |
| Speed | HRV | c_5’_ | 0.07 | 0.05 | 1.47 | 0.14 | -0.02 – 0.16 |
| Speed | HRV^2^ | c_6’_ | -0.01 | 0.04 | -0.21 | 0.84 | -0.07 – 0.08 |
| PSMD | SSBP | a_1_ | -0.02 | 0.05 | -0.43 | 0.66 | -0.11 – 0.07 |
| PSMD | SSBPP^2^ | a_2_ | 0.00 | 0.04 | -0.04 | 0.97 | -0.08 – 0.07 |
| PSMD | PP | a_3_ | 0.31 | 0.04 | 6.90 | **<0.01** | 0.22 – 0.40 |
| PSMD | PP^2^ | a_4_ | 0.09 | 0.04 | 2.42 | **0.02** | 0.02 – 0.16 |
| PSMD | HRV | a_5_ | -0.35 | 0.04 | -8.78 | **<0.01** | -0.44 – -0.28 |
| PSMD | HRV^2^ | a_6_ | -0.01 | 0.04 | -0.33 | 0.74 | -0.08 – 0.07 |
| Speed | PSMD | b_1_ | -0.20 | 0.06 | -3.37 | **<0.01** | -0.30 – -0.08 |
| PSMD | Sex |  | 0.12 | 0.07 | 3.27 | **<0.01** | 0.09 – 0.39 |
| PSMD | Head Motion |  | 0.13 | 0.04 | 3.16 | **<0.01** | 0.07 – 0.24 |
| Speed | Sex |  | 0.07 | 0.07 | 1.75 | 0.08 | -0.01 – 0.27 |
| Speed | Age |  | -0.49 | 0.06 | -8.16 | **<0.01** | -0.59 – -0.36 |
| Total Absolute Mediation of  SSBP + SSBP^2^ |  | (a_1_ x b) +  (a_2_ x b) | 0.00 | 0.01 | 0.48 | 0.63 | 0.00 – 0.04 |
| TAM of PP + PP^2^ |  | (a_3_ x b) +  (a_4_ x b) | 0.08 | 0.03 | 2.91 | **<0.01** | 0.03 – 0.13 |
| TAM of HRV + HRV^2^ |  | (a_5_ x b) +  (a_6_ x b) | 0.07 | 0.02 | 2.90 | **<0.01** | 0.03 – 0.12 |

Abbreviations: HRV, heart rate variability; SSBP, steady state blood pressure; PP, pulse pressure; PSMD, peak width of skeletonized mean diffusivity; TAM – Total Absolute Mediation.

Table S18. Structural Equation Model 1A using observed variables of pulse pressure. Significant effects where confidence intervals do not cross zero are in bold.

| Dependent variable | Predictor variable | Label | Standard β | Standard Error | z | p | Confidence Intervals |
| --- | --- | --- | --- | --- | --- | --- | --- |
| Speed | PP | c_1_’ | -0.107 | 0.045 | -2.36 | **0.02** | -0.19 – -0.02 |
| Speed | PP^2^ | c_2_^’^ | -0.018 | 0.043 | -0.431 | 0.67 | -0.10 – 0.07 |
| PSMD | PP | a_1_ | 0.449 | 0.04 | 11.23 | **<0.01** | 0.37 – 0.53 |
| PSMD | PP^2^ | a_2_ | 0.111 | 0.038 | 2.922 | **<0.01** | 0.04 – 0.19 |
| Speed | PSMD | b_1_ | -0.549 | 0.049 | -11.291 | **<0.01** | -0.63 - -0.46 |
| PSMD | Sex |  | 0.139 | 0.08 | 1.744 | 0.08 | -0.02 – 0.30 |
| PSMD | Head Motion |  | 0.145 | 0.047 | 3.077 | **<0.01** | 0.08 – 0.26 |
| Speed | Sex |  | -0.107 | 0.045 | -2.36 | **0.02** | 0.19 – 0.48 |
| TAM of  PP + PP^2^ |  | (a_1_ x b) +  (a_2_ x b) | 0.31 | 0.04 | 7.3 | **<0.01** | 0.23 – 0.40 |

Abbreviations: PP, pulse pressure; PSMD, peak width of skeletonized mean diffusivity; TAM – Total Absolute Mediation.

Table S19. Structural Equation Model 1B using observed variables of pulse pressure. Significant effects where confidence intervals do not cross zero are in bold.

| Dependent variable | Predictor variable | Label | Standard β | Standard Error | z | p | Confidence Intervals |
| --- | --- | --- | --- | --- | --- | --- | --- |
| Speed | PP | c_1’_ | 0.006 | 0.042 | 0.148 | 0.88 | -0.08 – 0.09 |
| Speed | PP^2^ | c_2’_ | -0.008 | 0.038 | -0.204 | 0.84 | -0.08 – 0.07 |
| PSMD | PP | a_1_ | 0.449 | 0.04 | 11.229 | **<0.01** | 0.37 – 0.53 |
| PSMD | PP^2^ | a_2_ | 0.111 | 0.038 | 2.923 | **<0.01** | 0.04 – 0.19 |
| Speed | PSMD | b_1_ | -0.172 | 0.064 | -2.706 | **<0.01** | -0.29 – -0.05 |
| PSMD | Sex |  | 0.139 | 0.08 | 1.743 | 0.08 | -0.02 – 0.30 |
| PSMD | Head Motion |  | 0.145 | 0.047 | 3.078 | **<0.01** | 0.08 – 0.27 |
| Speed | Sex |  | 0.196 | 0.071 | 2.769 | **<0.01** | 0.06 – 0.34 |
| Speed | Age |  | -0.552 | 0.056 | -9.848 | **<0.01** | -0.67 – -0.45 |
| TAM of  PP + PP^2^ |  | (a_1_ x b) +  (a_2_ x b) | 0.10 | 0.04 | 2.51 | **<0.01** | 0.03 – 0.18 |

Abbreviations: PP, pulse pressure; PSMD, peak width of skeletonized mean diffusivity; TAM – Total Absolute Mediation.

Table S20. Likelihood ratio test results on Structural Equation Model 1B using observed variables, comparing the full model to a version where the path to Age was essentially removed, by constraining it to be equal to zero.

| Model | DF | AIC | BIC | Chi-sq | p |
| --- | --- | --- | --- | --- | --- |
| SEM 1b, full model | 2 | 8, 678 | 8, 789 | 377.65 |  |
| SEM 1b, constraining Age | 3 | 8, 777 | 8, 882 | 427.22 | <0.001 |

Abbreviations: AIC, Akiake Information Criterion; BIC, Bayesian Information Criterion; DF, degrees of freedom.

Table S21. Structural Equation Model 1C using observed variables of pulse pressure. Significant effects where confidence intervals do not cross zero are in bold.

| Dependent variable | Predictor  variable | label | Standard β | Standard Error | z | p | Confidence  intervals |
| --- | --- | --- | --- | --- | --- | --- | --- |
| Speed | PP | c_1’_ | 0.017 | 0.045 | 0.365 | 0.72 | -0.07 – 0.10 |
| Speed | PP^2^ | c_2’_ | -0.006 | 0.038 | -0.156 | 0.88 | -0.08 – 0.07 |
| PSMD | PP | a_1_ | 0.449 | 0.04 | 11.23 | **<0.01** | 0.37 – 0.53 |
| PSMD | PP^2^ | a_2_ | 0.111 | 0.038 | 2.923 | **<0.01** | 0.04 – 0.18 |
| Speed | PSMD | b_1_ | -0.153 | 0.067 | -2.297 | **0.02** | -0.28 – -0.02 |
| PSMD | Sex |  | 0.139 | 0.08 | 1.743 | 0.08 | -0.02 – 0.29 |
| PSMD | Head Motion |  | 0.145 | 0.047 | 3.078 | **<0.01** | 0.08 – 0.26 |
| Speed | Sex |  | 0.184 | 0.072 | 2.543 | **<0.01** | 0.04 – 0.33 |
| Speed | Age |  | -0.571 | 0.061 | -9.435 | **<0.01** | -0.70 – -0.46 |
| Speed | Age^2^ |  | -0.036 | 0.038 | -0.952 | 0.34 | -0.11 – 0.04 |
| TAM of PP + PP^2^ |  | (a_1_ x b) +  (a_2_ x b) | 0.09 | 0.04 | 2.20 | **0.03** | 0.01 – 0.17 |

Abbreviations: PP, pulse pressure; PSMD, peak width of skeletonized mean diffusivity; TAM – Total Absolute Mediation.

Table S22. Likelihood ratio test results comparing Structural Equation Model 1C using observed variables of pulse pressure, comparing the full model to a version where the path to Age^2^ was essentially removed, by constraining it to be equal to zero.

| Model | DF | AIC | BIC | Chi-sq | p |
| --- | --- | --- | --- | --- | --- |
| SEM 1c, full model | 3 | 10, 069 | 10, 208 | 386.08 |  |
| SEM 1c, constraining Age^2^ | 4 | 10, 068 | 10, 203 | 387.08 | 0.32 |

Abbreviations: AIC, Akiake Information Criterion; BIC, Bayesian Information Criterion; DF, degrees of freedom.

Table S23. Structural Equation Model 1D using observed variables of pulse pressure and steady state blood pressure. Significant effects where confidence intervals do not cross zero are in bold

| Dependent variable | Predictor  variable | label | Standard β | Standard Error | z | p | Confidence Intervals |
| --- | --- | --- | --- | --- | --- | --- | --- |
| Speed | SSBP | c_1’_ | 0.032 | 0.041 | 0.783 | 0.43 | -0.05 – 0.11 |
| Speed | SSBPP^2^ | c_2’_ | 0.025 | 0.036 | 0.711 | 0.48 | -0.04 – 0.10 |
| Speed | PP | c_3’_ | -0.009 | 0.048 | -0.183 | 0.85 | -0.10 – 0.09 |
| Speed | PP^2^ | c_4’_ | -0.014 | 0.041 | -0.355 | 0.72 | -0.09 – 0.06 |
| Speed | HRV | c_5’_ | 0.084 | 0.045 | 1.871 | 0.06 | 0.002 – 0.18 |
| Speed | HRV^2^ | c_6’_ | 0.024 | 0.036 | 0.674 | 0.50 | -0.05 – 0.09 |
| PSMD | SSBP | a_1_ | -0.016 | 0.05 | -0.322 | 0.75 | -0.12 – 0.08 |
| PSMD | SSBPP^2^ | a_2_ | -0.007 | 0.042 | -0.158 | 0.88 | -0.08 – 0.07 |
| PSMD | PP | a_3_ | 0.34 | 0.048 | 7.092 | **<0.01** | 0.25 – 0.44 |
| PSMD | PP^2^ | a_4_ | 0.084 | 0.038 | 2.222 | **0.03** | 0.02 – 0.16 |
| PSMD | HRV | a_5_ | -0.331 | 0.043 | -7.623 | **<0.01** | -0.42 – -0.25 |
| PSMD | HRV^2^ | a_6_ | 0 | 0.041 | -0.001 | 1.00 | -0.08 – 0.08 |
| Speed | PSMD | b_1_ | -0.176 | 0.063 | -2.777 | **<0.01** | -0.29 – -0.05 |
| PSMD | Sex |  | 0.189 | 0.078 | 2.408 | **0.02** | 0.04 – 0.34 |
| PSMD | Head Motion |  | 0.123 | 0.046 | 2.654 | **<0.01** | 0.06 – 0.24 |
| Speed | Sex |  | 0.195 | 0.073 | 2.679 | **<0.01** | -0.05 – 0.34 |
| Speed | Age |  | -0.501 | 0.062 | -8.064 | **<0.01** | -0.63 – -0.38 |
| Total Absolute Mediation of  SSBP + SSBP^2^ |  | (a_1_ x b) +  (a_2_ x b) | 0.004 | 0.009 | 0.445 | 0.66 | 0.00 – 0.04 |
| TAM of PP + PP^2^ |  | (a_3_ x b) +  (a_4_ x b) | 0.075 | 0.03 | 2.502 | **0.01** | 0.02 – 0.14 |
| TAM of HRV + HRV^2^ |  | (a_5_ x b) +  (a_6_ x b) | 0.058 | 0.025 | 2.312 | **0.02** | 0.02 – 0.12 |

Abbreviations: HRV, heart rate variability; SSBP, steady state blood pressure; PP, pulse pressure; PSMD, peak width of skeletonized mean diffusivity; TAM – Total Absolute Mediation.

Table S24. Likelihood ratio test results comparing Structural Equation Model 2A, where no paths vary with Age, to Structural Equation Model 2B, where paths ‘a1’ and ‘a2’ vary across three age groups: Young (18-44 years, n=190), Middle (44-65 years, n=190) and Old (65-87 years, n=190).

| Model | DF | AIC | BIC | Chi-sq | p |
| --- | --- | --- | --- | --- | --- |
| SEM 2a: no paths vary across Age groups | 13 | 7, 062 | 7, 279 | 10.19 |  |
| SEM 2b: ‘a_1_’ and ‘a_2_’ vary across Age groups | 9 | 7, 068 | 7, 302 | 7.82 | 0.67 |

Abbreviations: AIC, Akiake Information Criterion; BIC, Bayesian Information Criterion; DF, degrees of freedom.

Table S25. Likelihood ratio test results on Structural Equation Model 3A, comparing the full model to a version where the ‘b_2_’ path between processing speed and the ability discrepancy was essentially removed, by constraining it to be equal to zero.

| Model | DF | AIC | BIC | Chi-sq | p |
| --- | --- | --- | --- | --- | --- |
| SEM 3a, full model | 6 | 11, 019 | 11, 149 | 366 |  |
| SEM 3a, constraining ‘b_2_’ | 7 | 11, 021 | 11, 147 | 370 | 0.04 |

Abbreviations: AIC, Akiake Information Criterion; BIC, Bayesian Information Criterion; DF, degrees of freedom.

Table S26. Structural Equation Model 3A. Significant effects where confidence intervals do not cross zero are in bold.

| Dependent variable | Predictor  variable | label | Standard β | Standard Error | z | p | Confidence  Intervals |
| --- | --- | --- | --- | --- | --- | --- | --- |
| Discrepancy | PP | c_1’_ | -0.01 | 0.04 | -0.24 | 0.81 | -0.09 – 0.07 |
| Discrepancy | PP^2^ | c_2’_ | -0.02 | 0.03 | -0.54 | 0.59 | -0.08 – 0.04 |
| PSMD | PP | a_1_ | 0.42 | 0.04 | 11.21 | **<0.01** | 0.35 – 0.50 |
| PSMD | PP^2^ | a_2_ | 0.11 | 0.04 | 3.07 | **<0.01** | 0.04 – 0.18 |
| Speed | PSMD | b_1_ | -0.20 | 0.06 | -3.31 | **<0.01** | -0.29 – -0.07 |
| Discrepancy | Speed | b_2_ | -0.08 | 0.04 | -2.04 | **0.04** | -0.16 – 0.00 |
| PSMD | Sex |  | 0.10 | 0.08 | 2.58 | **0.01** | 0.05 – 0.34 |
| PSMD | Head Motion |  | 0.17 | 0.05 | 3.83 | **<0.01** | 0.11 – 0.29 |
| Speed | Sex |  | 0.07 | 0.07 | 1.90 | 0.06 | 0.00 – 0.25 |
| Speed | Age |  | -0.55 | 0.05 | -10.32 | **<0.01** | -0.63 – -0.42 |
| Discrepancy | Sex |  | -0.02 | 0.06 | -0.57 | 0.57 | -0.16 – 0.08 |
| Discrepancy | Age |  | 0.63 | 0.05 | 13.86 | **<0.01** | 0.54 – 0.72 |
| TAM of PP + PP^2^ |  | (a_1_ x b_1_ x b_2_) +  (a_2_ x b_1_ x b_2_) | 0.01 | 0.01 | 1.76 | 0.08 | 0.00 – 0.02 |

Abbreviations: PP, pulse pressure; PSMD, peak width of skeletonized mean diffusivity; TAM – Total Absolute Mediation.

Table S27. Likelihood ratio test results on Structural Equation Model 3B, comparing the full model to a version where the ‘b_2_’ path between processing speed and fluid intelligence was essentially removed, by constraining it to be equal to zero.

| Model | DF | AIC | BIC | Chi-sq | p |
| --- | --- | --- | --- | --- | --- |
| SEM 3b, full model | 6 | 10, 950 | 11, 080 | 373.74 |  |
| SEM 3b, constraining ‘b_2_’ | 7 | 11, 042 | 11, 167 | 467.75 | <0.001 |

Abbreviations: AIC, Akiake Information Criterion; BIC, Bayesian Information Criterion; DF, degrees of freedom.

Table S28. Structural Equation Model 3B. Significant effects where confidence intervals do not cross zero are in bold.

| Dependent variable | Predictor  variable | label | Standard β | Standard Error | z | p | Confidence  Intervals |
| --- | --- | --- | --- | --- | --- | --- | --- |
| Fluid | PP | c_1’_ | -0.06 | 0.03 | -1.71 | 0.09 | -0.13 – 0.01 |
| Fluid | PP^2^ | c_2’_ | 0.03 | 0.03 | 0.94 | 0.35 | -0.03 – 0.08 |
| PSMD | PP | a_1_ | 0.42 | 0.04 | 11.21 | **<0.01** | 0.35 – 0.50 |
| PSMD | PP^2^ | a_2_ | 0.11 | 0.04 | 3.07 | **<0.01** | 0.04 – 0.18 |
| Speed | PSMD | b_1_ | -0.20 | 0.06 | -3.31 | **<0.01** | -0.29 – -0.07 |
| Fluid | Speed | b_2_ | 0.38 | 0.04 | 9.12 | **<0.01** | 0.31 – 0.48 |
| PSMD | Sex |  | 0.10 | 0.08 | 2.58 | **0.01** | 0.05 – 0.34 |
| PSMD | Head Motion |  | 0.17 | 0.05 | 3.83 | **<0.01** | 0.11 – 0.29 |
| Speed | Sex |  | 0.07 | 0.07 | 1.90 | 0.06 | 0.00 – 0.25 |
| Speed | Age |  | -0.55 | 0.05 | -10.32 | **<0.01** | -0.63 – -0.42 |
| Fluid | Sex |  | 0.07 | 0.06 | 2.41 | **0.02** | 0.02 – 0.26 |
| Fluid | Age |  | -0.38 | 0.04 | -8.75 | **<0.01** | -0.46 – -0.29 |
| TAM of PP + PP^2^ |  | (a_1_ x b_1_ x b_2_) +  (a_2_ x b_1_ x b_2_) | 0.04 | 0.01 | 2.77 | **0.01** | 0.01 – 0.07 |

Abbreviations: PP, pulse pressure; PSMD, peak width of skeletonized mean diffusivity; TAM – Total Absolute Mediation.

Table S29. Likelihood ratio test results comparing Structural Equation Model 3B with the expected and reversed orders of variables, excluding covariats of no interest. No p-value is given when models have the same degrees of freedom.

| Model | DF | AIC | BIC | Chi-sq |
| --- | --- | --- | --- | --- |
| Expected order:  Speed (b_2_) – Fluid | 3 | 7, 328 | 7, 380 | 43.59 |
| Reversed order:  Fluid (b_2_) – Speed | 3 | 7, 346 | 7, 398 | 62.39 |

Abbreviations: AIC, Akiake Information Criterion; BIC, Bayesian Information Criterion; DF, degrees of freedom; PP, pulse pressure; PSMD, peak width of skeletonized mean diffusivity.

Table S30. Likelihood ratio test results comparing Structural Equation Model 3B with the expected and reversed orders of variables, including covariates of no interest. No p-value is given when models have the same degrees of freedom.

| Model | DF | AIC | BIC | Chi-sq |
| --- | --- | --- | --- | --- |
| Expected order:  Speed (b_2_) – Fluid | 6 | 10, 950 | 11, 080 | 373.74 |
| Reversed order:  Fluid (b_2_) – Speed | 6 | 10, 952 | 11, 082 | 375.77 |

Abbreviations: AIC, Akiake Information Criterion; BIC, Bayesian Information Criterion; DF, degrees of freedom; PP, pulse pressure; PSMD, peak width of skeletonized mean diffusivity.

## Supplemental Figures


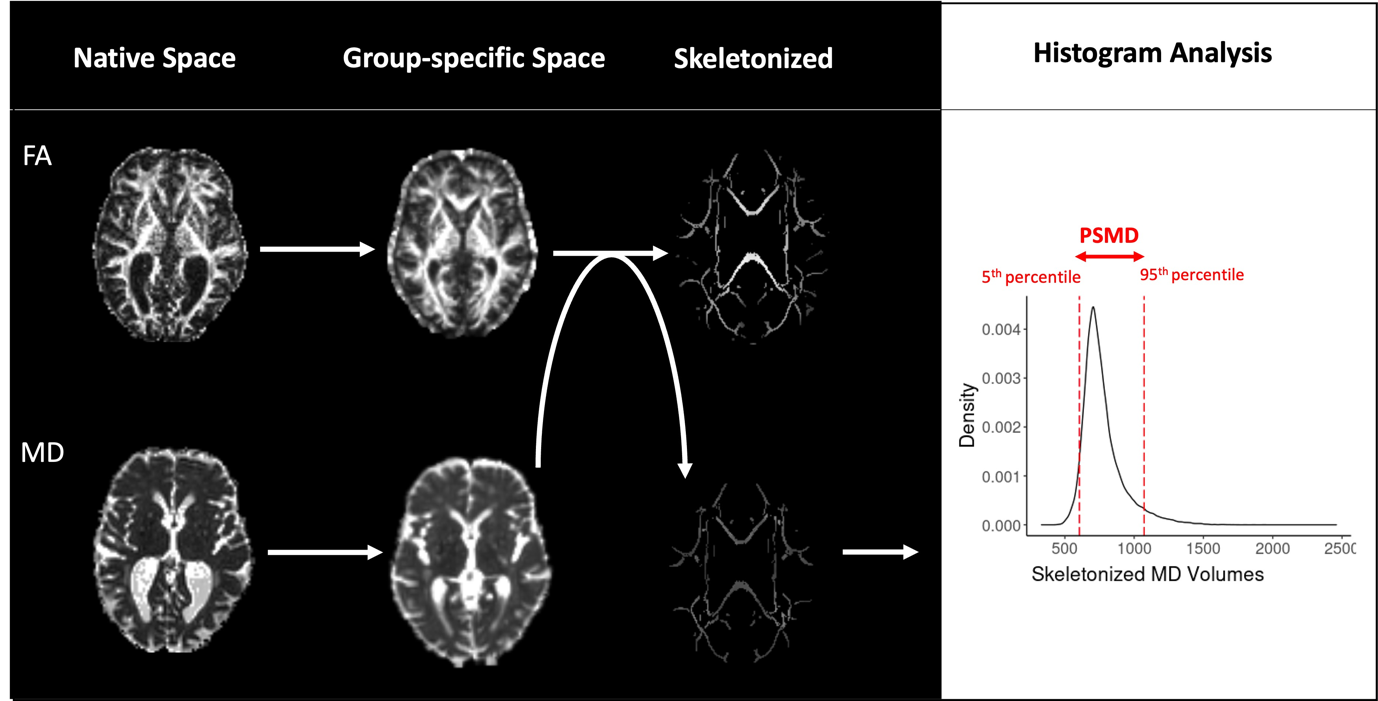


Figure S1. An illustration of the main steps in the procedure to calculate the peak width of skeletonised mean diffusivity (PSMD). The diffusion tensor imaging metrics of fractional anisotropy (FA) and mean diffusivity (MD) values (mm^2^/s) were normalised to MNI space. Normalised FA values were then projected onto a skeleton template. The skeletonization projection parameters were next applied to the normalised MD values. The skeletonized and masked MD values were input to histogram analysis (right). The difference between the 5^th^ and 95^th^ percentiles (red dashed lines) was calculated, to give PSMD. Higher PSMD indicates greater heterogeneity in white matter integrity, potentially indicating diffuse white matter damage.


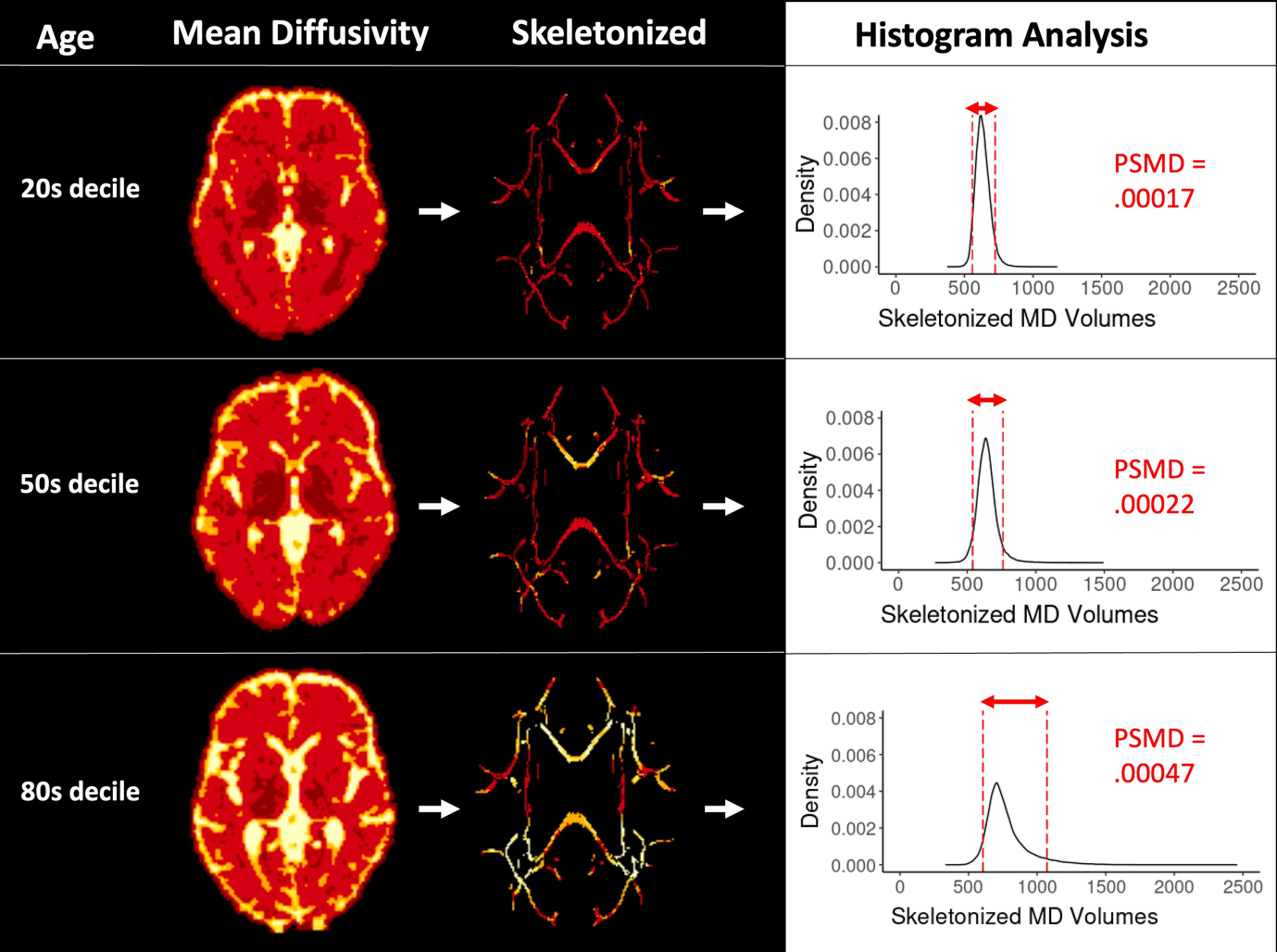


Figure S2. An illustration of the main steps in calculating of the peak width of skeletonised mean diffusivity (PSMD) for three example participants: individuals from the 20s, 50s and 80s age deciles, including males and females. The mean diffusivity images (left), here normalised to standard space, are coloured red to yellow indicating low to high mean diffusivity values (mm^2^/s). High diffusivity is shown in yellow. In the skeletonized images (middle), a greater range of colours within an image suggests a greater range of mean diffusivity values. The distribution and density of mean diffusivity values were plotted on histograms (right). The difference between the 5^th^ and 95^th^ percentiles (red dashed lines) was used to calculate PSMD. Higher PSMD indicates non-uniform white matter integrity. Here, PSMD values were lowest in the youngest participant and highest in the oldest participant.


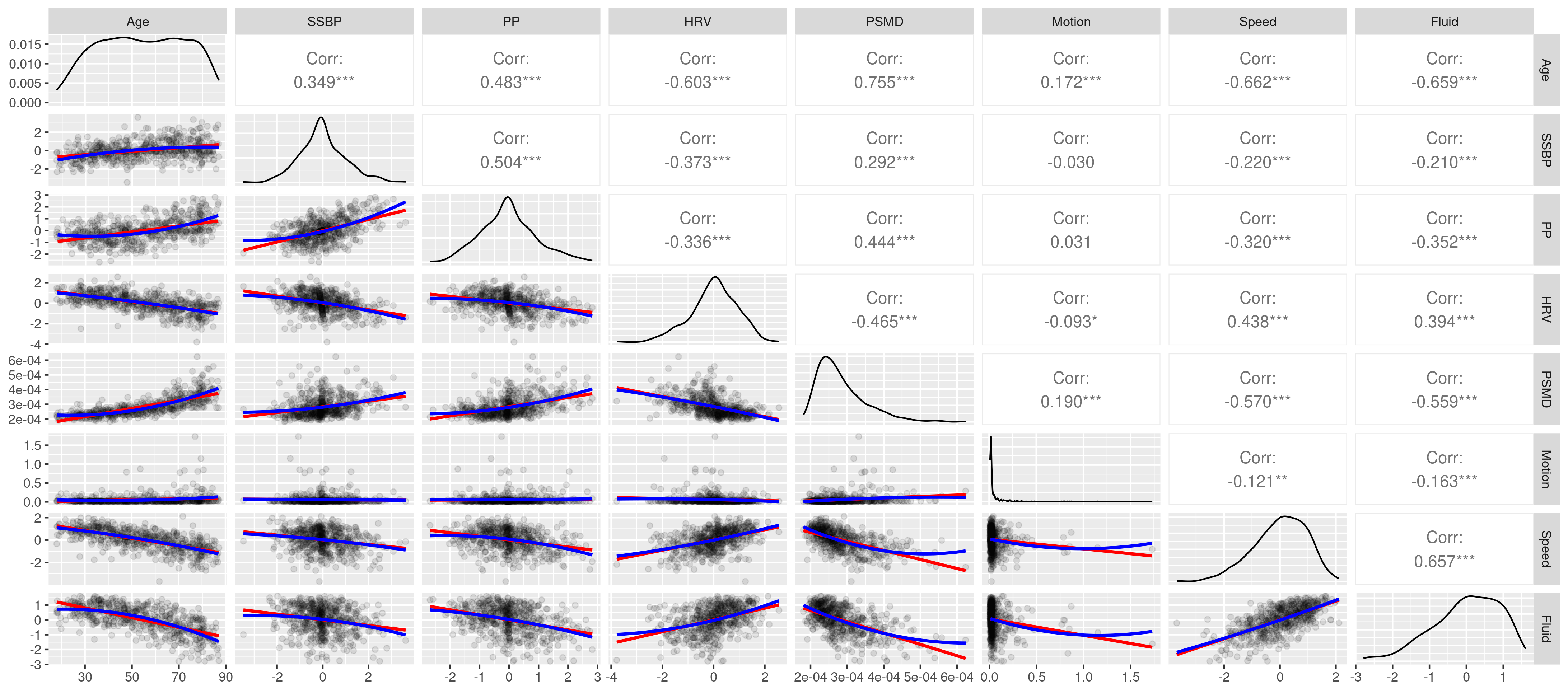


Figure S3. Scatter plots (lower left), distributions (leading diagonal) and Pearson correlations (upper right) for age, latent vascular factors, PSMD, head motion, processing speed and fluid intelligence (n=564). Scatter plots show linear (red) and quadratic (blue) associations and data intensity (greyscale). Stars indicate increasing significance on the correlations: ***, p<0.001; **, p<0.01; *, p<0.05.Abbreviations: SS BP, steady state blood pressure latent factor; Corr, correlation coefficient; HRV, heart rate variability latent factor; PSMD, peak width of skeletonised mean diffusivity; PP, pulse pressure latent factor.


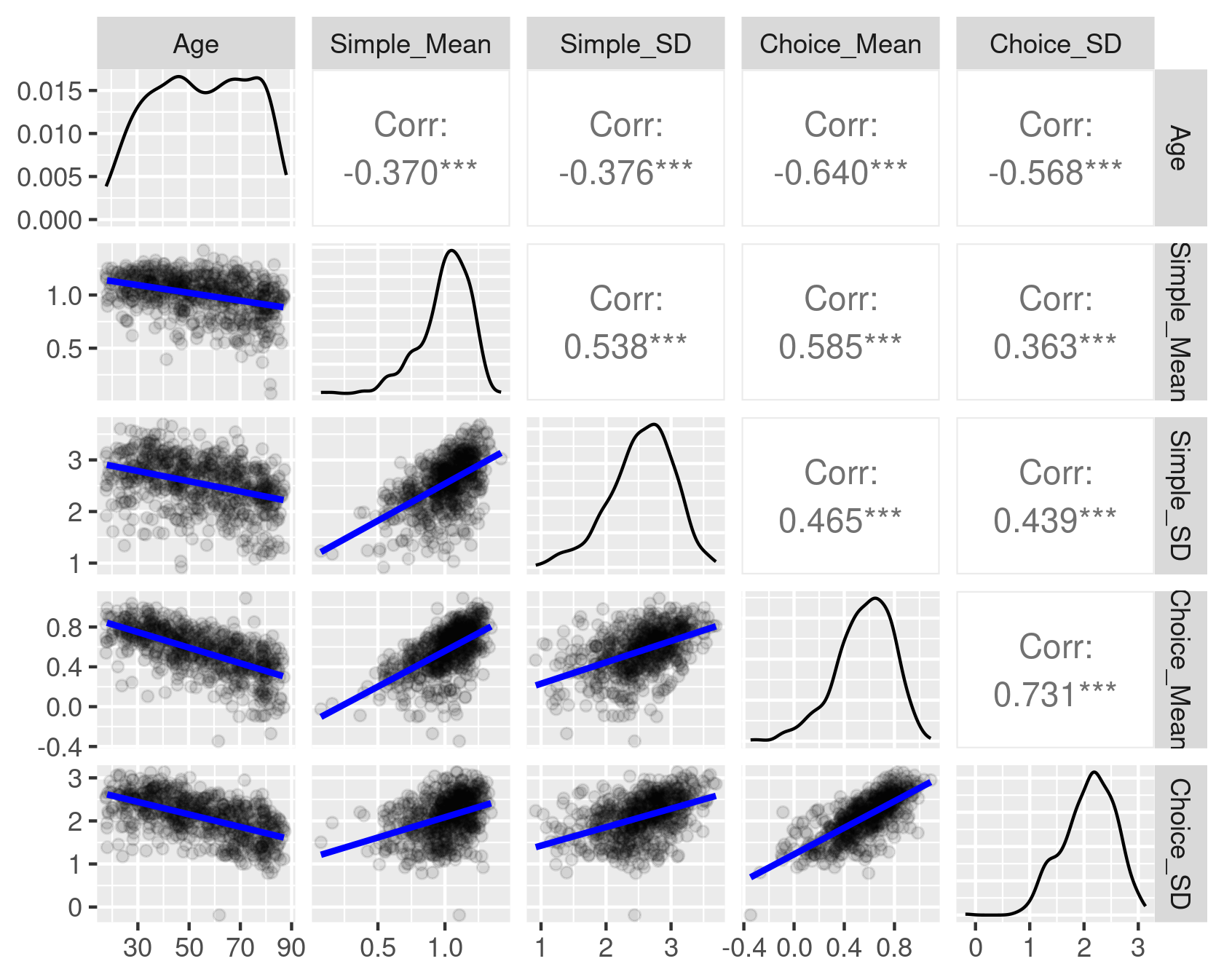


Figure S4. Scatter plots (lower left), distributions (leading diagonal) and Pearson correlations (upper right) for age and cognitive observed variables. Scatter plots show linear associations (blue) and data intensity (greyscale). Stars indicate increasing significance on the correlations: ***, p<0.001; **, p<0.01; *, p<0.05. Abbreviations: Choice_M, choice task mean; Choice_SD, choice task standard deviation; Corr, correlation coefficient; Simple_M, simple task mean; Simple_SD, simple task standard deviation.


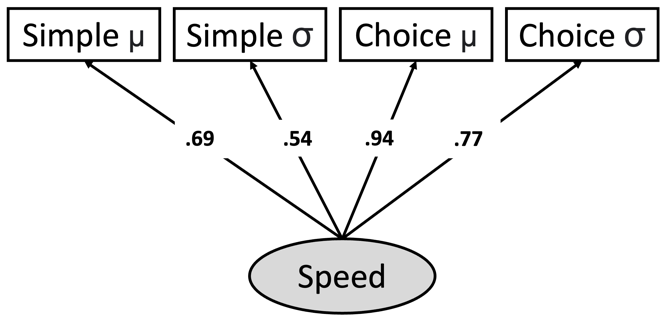


Figure S5. The one-factor Confirmatory Factor Analysis model of processing speed (n=664).


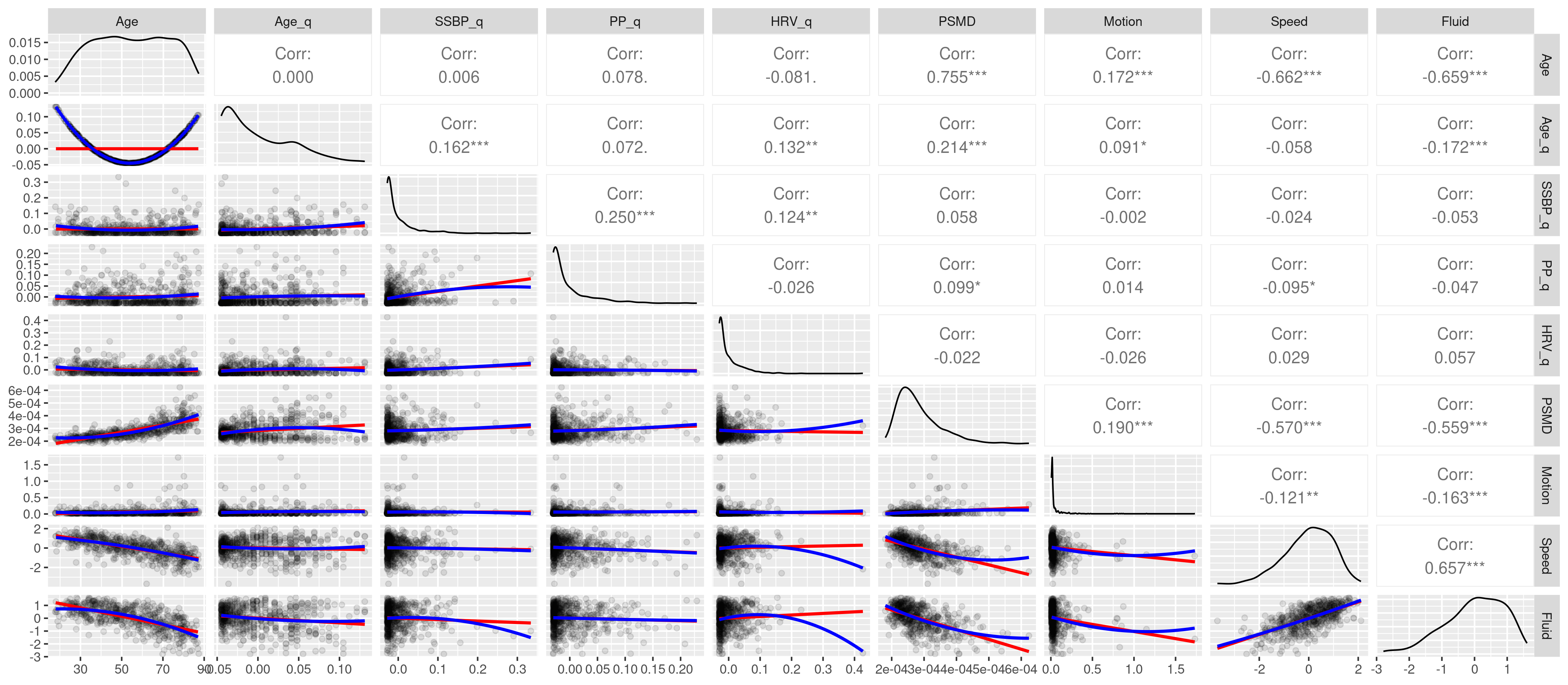


Figure S6. Scatter plots (lower left), distributions (leading diagonal) and Pearson correlations (upper right) for age, quadratic age, quadratic latent vascular factors, PSMD, head motion, processing speed and fluid intelligence (n=564). Scatter plots show linear (red) and quadratic (blue) associations and data intensity (greyscale). Stars indicate increasing significance on the correlations: ***, p<0.001; **, p<0.01; *, p<0.05. Variables named “xx_q” are in quadratic form.


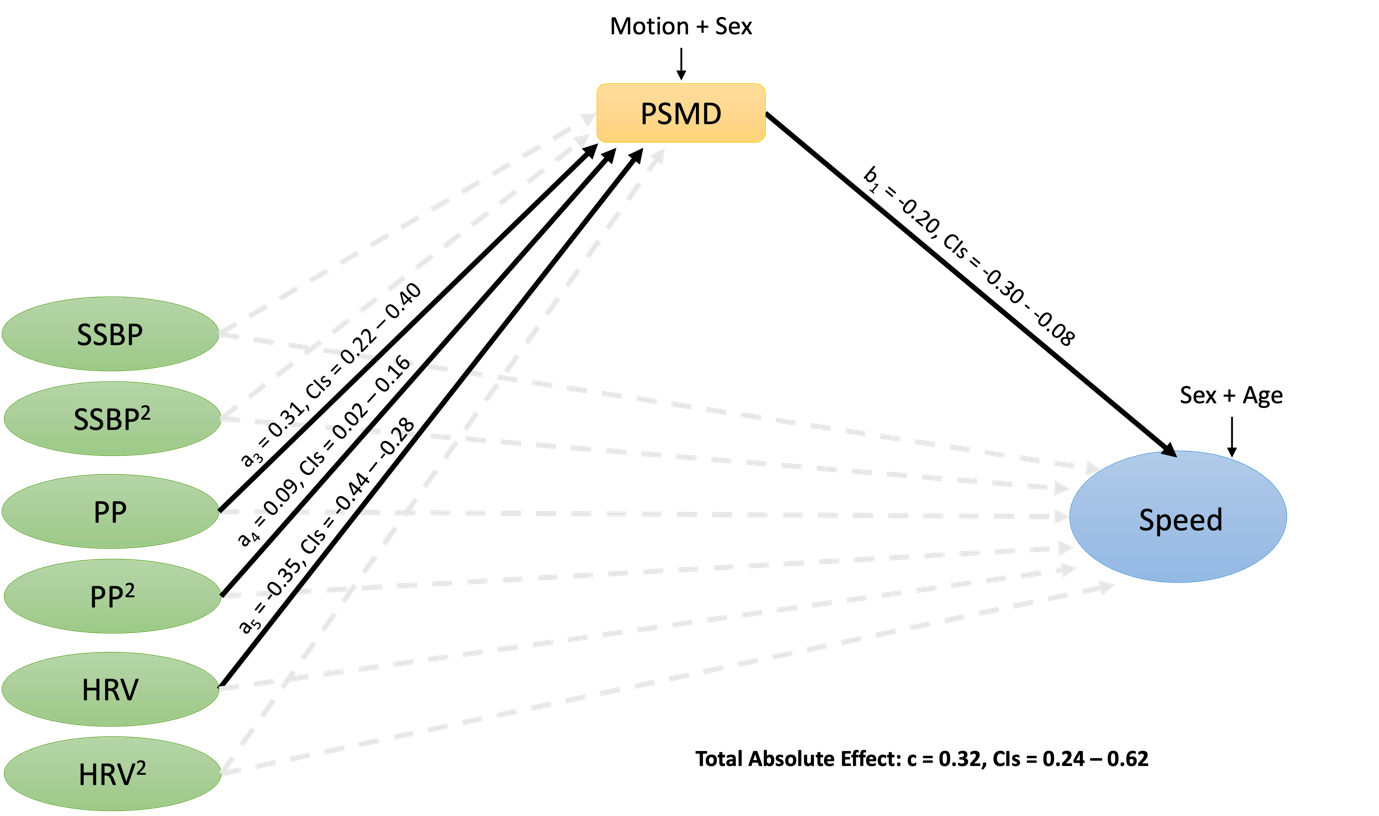


Figure S7. Structural Equation Models 1D (n=570). Standardized betas shown for significant paths only, for clarity. Dashed lines represent insignificant results. Squares represent manifest variables and circles represent latent variables. Abbreviations: HRV, heart rate variability; SSBP, steady state blood pressure; PP, pulse pressure; PSMD, peak width of skeletonized mean diffusivity.
